# Astrocyte CB_1_ receptors drive blood-brain barrier disruption in central nervous system inflammatory disease

**DOI:** 10.1186/s12974-026-03708-3

**Published:** 2026-01-29

**Authors:** Teresa Colomer, Ana Bernal-Chico, Ester Sánchez-Martín, Alvaro Moreno-García, Andrés Mateo Baraibar, Aitziber Uribe-Irusta, Ander Iriarte-Sarria, Sandra Beriain, Urszula Skupio, Charlotte Gatuingt-Chasseriaud, Delphine Gonzales, Guillaume Laplagne, Román Serrat, Isabel Pidal-Ladrón de Guevara, Carlos Matute, Diego Clemente, Vanja Tepavcevic, Ignacio Fernández-Moncada, Candice Chapouly, Giovanni Marsicano, Susana Mato

**Affiliations:** 1 Department of Neurosciences, University of the Basque Country UPV/EHU, Leioa, 48940 Spain; 2 Achucarro Basque Center for Neuroscience, Leioa, 48940 Spain; 3 Neuroimmunology Group, Biobizkaia Health Research Institute, Barakaldo, 48903 Spain; 4 University of Bordeaux, INSERM, Neurocentre Magendie, U1215, Bordeaux, 33000 France; 5 University of Bordeaux, INRAE, Bordeaux INP, NutriNeurO, UMR 1286, Bordeaux, 33000 France; 6 University of Bordeaux, INSERM, BMC, U1034, Pessac, 33600 France; 7 Neuroimmuno-Repair Group, Hospital Nacional de Parapléjicos (SESCAM), Toledo, 45071 Spain; 8 Castile-La Mancha Health Research Institute (IDISCAM), Toledo, 45071 Spain; 9 Centro de Investigación Biomédica en Red sobre Enfermedades Neurodegenerativas (CIBERNED), Madrid, 28029 Spain; 10 Laboratory of Comparative and Regenerative Neurobiology, Cavanilles Institute of Biodiversity and Evolutionary Biology, University of Valencia, Paterna, 46980 Spain; 11 Department of Cell Biology, Functional Biology and Physical Anthropology, University of Valencia, Burjassot, 46100 Spain

**Keywords:** Multiple sclerosis, Astrocyte, CB_1_ receptors, Experimental autoimmune encephalomyelitis, Blood-brain barrier, Remyelination

## Abstract

Reactive astrocytes shape central nervous system (CNS) inflammation and participate in myelin damage and repair mechanisms in multiple sclerosis (MS). Through the activation of cannabinoid CB_1_ receptors (CB_1_R) expressed by neurons and oligodendrocyte lineage cells, endocannabinoid signaling restricts neurodegeneration and promotes remyelination in preclinical MS models. However, despite accumulating evidence that supports cell-specific roles for CB_1_R in brain physiology and pathology, the implications of astrocyte CB_1_R signaling in MS initiation and progression remain uncertain. Using complementary in vivo disease models, here we investigated the effects of targeted genetic deletion of astrocyte CB_1_R on the expression of MS-like pathology in mice. Interestingly, astrocyte-specific deletion of CB_1_R reduced demyelinating neuropathology, attenuated astrocyte reactivity and improved clinical deficits during the time-course of experimental autoimmune encephalomyelitis (EAE). Mice with astrocyte CB_1_R inactivation displayed unaltered oligodendrocyte populations both in EAE plaques and in lysolecithin-induced remyelinating spinal cord lesions, likely excluding that CB_1_R expressed by astroglial cells modulate myelin repair processes. Conversely, inactivation of CB_1_R in astrocytes restricted humoral and leukocyte parenchymal infiltration and reduced the expression of vascular effectors in EAE lesions. Finally, loss of blood-brain barrier (BBB) function induced by cortical microinjection of VEGF-A was less severe in astrocyte CB_1_R null mice. These results show that astrocyte CB_1_R signaling constitutes a significant pro-inflammatory mechanism in experimental MS and bring to light a deleterious role for endocannabinoid-mediated modulation of astroglial cells with potential implications in the etiopathology and therapy of neuroinflammatory disorders.

**Supplementary Information:**

The online version contains supplementary material available at 10.1186/s12974-026-03708-3.

## Introduction

Multiple sclerosis (MS) is an immune-mediated inflammatory disease of the central nervous system (CNS) and one of the most prevalent neurological disorders leading to chronic disability among young adults [1]. The main pathological hallmark of MS is the formation of demyelinating lesions in the brain and spinal cord associated with neuroaxonal degeneration as primary substrate of the irreversible clinical deficits that characterize disease progression [2]. Focal lesions in MS are thought to be caused by the bidirectional interaction between peripheral immune cells that infiltrate into the CNS parenchyma, including T cells, B cells and myeloid cells, and activated resident immune cells, mainly astrocytes and microglia [3]. A subset of lesions in MS patients are characterized by a variable extent of remyelination suggested as a mechanism of neuroprotection and clinical recovery [4, 5]. At present, treatment strategies for clinical exacerbation in MS include almost exclusively immunotherapeutic drugs that target peripheral immune cells and their trafficking into the CNS. These pharmacological therapies lead to a substantial reduction in lesion formation and clinical relapse rate but do not prevent the progression of clinical disability.

Astrocytes support brain homeostasis and function through a plethora of mechanisms that include the modulation of synaptic transmission through the release of gliotransmitters, the metabolic assistance to neurons and oligodendrocytes, and the maintenance of the blood-brain barrier (BBB), among others. In recent years, complementary lines of evidence have shown that astrocytes adopt a wide spectrum of reactive states during neuroinflammation that confer these cells the potential to exacerbate damage or facilitate repair [6–8]. Aberrant astrocyte activation critically contributes to inflammatory lesion formation and progression in MS through the release of molecules that promote the loss of BBB integrity and the recruitment of peripheral immune cells [8–10]. However, astrocytes may also facilitate the differentiation of oligodendrocyte progenitor cells (OPCs) and support the survival of mature oligodendrocytes to ensure successful remyelination [11, 12]. An improved understanding of astrocyte regulatory mechanisms in neuroinflammatory contexts may thus provide novel therapeutic targets that reduce MS pathology and clinical severity during acute and progressive phases of the disease.

Cannabinoid type-1 receptors (CB_1_R) are among the most abundant G protein-coupled receptors in the mammalian brain and the primary molecular targets of endogenous cannabinoids - anandamide and 2-arachidonoylglycerol - and Δ^9^-tetrahydrocannabinol (THC), the main psychoactive component of the hemp plant *Cannabis sativa* [13]. Endocannabinoids acting on CB_1_R heterogeneously expressed in neuronal populations and glial cells modulate physiological brain functions through a wide variety of cellular processes and exhibit neuroprotective potential during CNS damage [14–16]. Pharmacological and genetic studies conducted so far have demonstrated that cannabinoid agents that potentiate CB_1_R-mediated signaling attenuate neurodegeneration and suppress neuroinflammation while promoting myelin repair in rodent models of MS [17–20]. However, the clinical efficacy of cannabinoid-based medications in MS is limited, and therapeutic optimization requires a better understanding of endocannabinoid and CB_1_R related networks in inflammatory demyelinating contexts [16, 21]. On mechanistic grounds, there is consensus that the neuronal population of CB_1_R provides neuroprotection from excitotoxic damage in rodent models of MS by limiting synaptic glutamate release [20, 22–24]. Experimental in vivo evidence from conditional mutant mice has also recently grounded the hypothesis that CB_1_R expressed by OPCs promote oligodendrocyte differentiation and favor remyelination in MS [25, 26]. However, the implications of CB_1_R in astrocytes during MS onset and progression have been largely neglected despite the fundamental contribution of these receptor populations as effectors of (endo)cannabinoid-mediated signaling in the brain [27, 28]. Indeed, the combination of high-resolution electron microscopy, electrophysiology, calcium imaging and cell-specific mutagenesis techniques has demonstrated, during the last decade, that astrocytes in the brain and spinal cord express functional CB_1_R in perisynaptic, perivascular, and mitochondrial compartments [29–33]. Accordingly, CB_1_R activity has arisen as crucial modulator of astrocyte-derived gliotransmitter release and metabolic supply [27, 28, 33] with emerging implications in neuroinflammatory disorders [34].

In this study, we aimed at disentangling the roles of astrocyte CB_1_R (aCB_1_R) signaling in MS neuropathology using mice where CB_1_R are selectively ablated in astroglial cells and a combination of rodent models that recapitulate autoimmune demyelination, BBB breakdown and myelin repair. Our results highlight that aCB_1_R exacerbate neurological disability during autoimmune demyelination by fostering BBB permeability and recruitment of peripheral immune cells towards lesion sites. These observations identify a previously unexpected disease-promoting role of aCB_1_R during CNS inflammatory lesion formation with potential implications in MS pathogenesis and therapy.

## Materials and methods

### Mice

All experiments were performed in accordance with the Guide for the Care and Use of Laboratory Animals (National Research Council Committee, 2011) and the European Communities Council Directive of 22 September 2010 (2010/63/EU74). Experiments were approved by the local ethical committees of the University of the Basque Country (approval numbers 2017140, 2020005 and 2022245) and the University of Bordeaux (approval numbers A33063098 and 16901). Inducible mutant mice of a C57BL/6 N background lacking CB_1_R in cells expressing the astrocytic marker glial fibrillary acidic protein GFAP (aCB_1_-KO) and aCB_1_-WT littermates were bred at the Neurocentre Magendie (Bordeaux, France). Cages were enriched and mice were maintained under standard conditions (food and water *ad libitum*; 12 h–12 h light-dark cycle). Experiments were performed during dark cycle (light off at 8:00 h a.m.). In vivo models were induced in female mice based on epidemiological evidence that MS affects 2–4 times more women than men [35]. The number of mice in each experimental group was similar. No statistical methods were used to predetermine sample size.

aCB_1_-KO mice were generated by crossing mice carrying LoxP sites flanking the coding region of the *Cnr1* gene (CB_1_^f/f^) [36] with GFAP-CreERT2 mice [37] using a three-step backcrossing procedure to obtain CB_1_^f/f; GFAP−CreERT2^ and CB_1_^f/f^ littermates, called aCB_1_-KO and aCB_1_-WT respectively. Deletion of the *Cnr1* gene was obtained in adult aCB_1_-KO mice (6–12 weeks of age) by daily intraperitoneal (i.p.) injections of tamoxifen (1 mg dissolved at 10 mg/mL in 90% sesame oil, 10% ethanol) for 8 days [29, 38] and corroborated using a PCR-based strategy with genomic DNA (Figure S1). Tamoxifen-treated aCB_1_-WT were used as controls. Mutant mice were used for experiments 3–4 weeks after the last tamoxifen injection.

### EAE model

Mice were immunized in the flank by subcutaneous (s.c.) injection of 200 µg MOG_35 − 55_ peptide (MEVGWYRSPFSRVVHLYRNGK) (Peptide Synthesis Core Facilities of the Pompeu Fabra University, Spain) in incomplete Freund’s adjuvant supplemented with 1.2 mg *Mycobacterium tuberculosis* H37Ra (Difco Laboratories). Pertussis toxin (500 ng; Calbiochem) was injected i.p. on the day of immunization and again at 2 days post-immunization (dpi). Body weight and motor symptoms were recorded daily and scored from 0 to 8 as follows: 0, no detectable changes in muscle tone and motor behavior; 1, flaccid tail; 2, paralyzed tail; 3, impairment or loss of muscle tone in hindlimbs; 4, hindlimb hemiparalysis; 5, complete hindlimb paralysis; 6, complete hindlimb paralysis and loss of muscle tone in forelimbs; 7, tetraplegia; and 8, moribund.

### Demyelinating lesion induction

Demyelinating lesions were induced by stereotaxic injection of 1% lysophosphatidylcholine (LPC; 0.5 µL; Sigma-Aldrich) diluted in sterile saline solution (0.9% NaCl) into the spinal cord [39]. Mice were anesthetized by i.p. injection of a ketamine (100 mg/Kg; Fatro) and xylazine (20 mg/Kg; Calier) cocktail. Two longitudinal incisions into the *longissimus dorsi* at each side of the vertebral column were performed and the muscle tissue covering the column was removed. Animals were placed into a stereotaxic frame and the intervertebral space of the 13th thoracic vertebra was exposed by removing the connective tissue. An incision into dura mater was performed using a 30-gauge needle and LPC was injected into the white matter of the *dorsal funiculus* at a rate of 0.5 µL/min via a Hamilton syringe attached to a glass micropipette using a stereotaxic micromanipulator. The lesion site was marked with sterile charcoal. Following LPC injection, the muscle sheaths were closed using 3/0 Monocryl and the wound was sutured with 4/0 silk. Buprenorphine (0.1 mg/Kg; Dechra) was subcutaneously administered as postoperative analgesic treatment. Mice were euthanized and processed for immunohistochemistry 14 days after the surgery.

### VEGF-A microinjection

Mice were anesthetized using a local injection of lidocaine (20 mg/mL) under the skull skin and a mix of air and isoflurane (3% for induction and 1.5% for support). Animals were placed into a stereotaxic apparatus and mouse VEGF-A_165_ (60 ng in 3 µL of 0.9% NaCl) or vehicle were delivered into the cerebral cortex at y = 1 mm caudal to Bregma, x = 2 mm, z = 1.5 mm as previously described [40]. Mice received a subcutaneous injection of buprenorphine (0.1 mg/Kg; Vetergesic) 30 min before surgery and again 8 h post-surgery to assure constant analgesia. Animals were sacrificed 48 h after VEGF-A_165_ injection and processed for immunohistochemistry.

### Surgery for AAV administration and fiber implantation

Mice were anesthetized with isoflurane and placed on a heating-pad to keep the body temperature at 37 °C. Eye dehydration was prevented by topical application of ophthalmic gel and analgesia was achieved by s.c. injection of buprenorphine (0.05 mg/Kg; Buprecare). The skin above the skull was shaved with a razor and disinfected with modified ethanol 70% and betadine before an incision was made. Mice were placed into a stereotaxic frame and injected with an AAV encoding the genetically encoded calcium indicator GCaMP6f under the GFAP promoter (AAV-9/2-GFAP-hHBbI/E-GCaMP6f-bGHp(A)) (ETH Zürich) to carry out fiber photometry imaging of calcium activity in astrocytes. Virus titers were between 10^10^ and 10^12^ genomic copies per mL. Stereotaxic injections were targeted to the mouse somatosensory cortex according to the following coordinates (from bregma): anterior-posterior − 1.5; medial-lateral ± 2.5; dorsal-ventral − 1.5. Viral particles (400–500 nL) were injected at a maximum rate of 100 nL/min using a glass pipette attached to a Nanojet III (Drummond, Broomall, USA). Following virus delivery, the syringe was left in place for 10 min before being slowly withdrawn from the brain. The optical fiber (400 μm diameter) was placed 250 μm above the injection site during the same surgical session. Mice were weighed daily and individuals that failed to return to their pre-surgery body weight were excluded from subsequent experiments. All animals were treated with tamoxifen 1 week after the surgery.

### Fiber photometry imaging

Freely moving mice were imaged after 3 days of handling habituation. The day of recording each mouse was placed in a rectangular chamber and its behavior recorded using a camera placed above the chamber. Baseline recordings of spontaneous astrocyte activity were made for 15 min every 1–2 days starting 3 days before MOG administration. The calcium signal evoked by sensory stimulation of the tail was assessed at the end of the baseline period.

Cortical astrocyte GCaMP6f was imaged in vivo using 470 and 405 nm LEDs. The emitted fluorescence is proportional to the calcium concentration for stimulation at 470 nm [41, 42]. The isosbestic 405 nm stimulation (UV light) was used in alternation with the blue light (470 nm) for analysis purposes as the fluorescence emitted after this stimulation is not calcium-dependent [43]. The GCaMP6f fluorescence from the astrocytes was collected with a sCMOS camera through an optic fiber divided in 2 sections: a short fiber implanted in the brain of the mouse and a long fiber (modified patchcord), both connected through a ferrule-ferrule (1.25 mm) connection. MATLAB program (Matlabworks) was used to synchronize each image recording made by the camera, and the GCaMP6s light excitation made by the LEDs (470 and 405 nm). The two wavelengths of 470 and 405 nm at a power of 0.1 mW were alternated at a frequency of 20 Hz each (40 Hz alternated light stimulations).

To calculate fluorescence due specifically to calcium fluctuations and to remove bleaching and movement artifacts, the isosbestic 405 nm signal was subtracted from the 470 nm calcium signal. Specifically, normalized fluorescence changes (∆*F*/*F*_*0*_) were calculated by subtracting the mean fluorescence (2 min sliding window average) from the fluorescence recorded by the fiber at each time point and dividing this value by the mean fluorescence (*F-F*_mean_)/*F*_mean_) using a customized Matlab software. Subsequently, the calcium independent isosbestic signal was subtracted from the raw signal emitted after the 470 nm excitation to eliminate unspecific fluorescence. The result is the global calcium signal (∆*F*/*F* (%) = ∆*F*_Ca_ - ∆*F*_iso_), that was used as an estimate of tonic activity of the astrocytes. Calcium transients were detected on the filtered trace using a threshold to identify them (2 median absolute deviation - MAD - of the entire trace). Amplitude was determined as the MAD of each studied period [44].

### Flow cytometry analysis of splenic populations

Fresh spleens were collected from aCB_1_-KO and aCB_1_-WT mice anesthetized with isoflurane and mechanically processed to obtain a single cell suspension, which was passed through a 40-µm filter (BD Biosciences), and washed with cold supplemented RPMI medium (Gibco, #1640) containing 2 mM L-Glutamine (Thermo Fisher), 10% fetal bovine serum (FBS) and 1% penicillin/streptomycin (P/S). Erythrocytes were lysed with 1 mL of ACK buffer (8.29 g/L NH_4_Cl, 1 g/L KHCO_3_, 1 mM EDTA in distilled H_2_O; pH 7.4) and the reaction was stopped with phosphate buffer saline (PBS). Splenocytes were recovered by centrifugation at 210 *x* g for 5 min, and 2 × 10^6^ cells were resuspended in 50 µL of staining buffer (PBS supplemented with 10% FBS, 25 mM HEPES buffer, 2% P/S). The Fc cell receptors were blocked with an anti-CD16/CD32 antibody (10 µg/mL; BD Biosciences; #553142) for 10 min at 4 °C. Cells were then labelled for 30 min at 4 °C in the dark with fluorochrome-conjugated antibodies targeting lymphoid (CD3e, CD4, CD8a, CD25, CD69 and CD19) and myeloid (CD11b, Ly6C, Ly6G, CD11c) markers at 0.025–0.2 µg/10^6^ cells (Table S1). Cells were rinsed with PBS, centrifuged at 210 *x* g for 5 min, resuspended in PBS and analyzed in a FACS Canto II cytometer (BD Biosciences). Data were processed using FlowJo 10.9.0 software (Tree Star Inc.). Fluorescence Minus One (FMO) controls were used to set gates and determine positive expression for each marker.

### Genotyping of the *Cnr1* allele

Genotyping of the *Cnr1* locus was performed following previously described procedures [36] with some modifications. Genomic DNA was extracted from forebrain and spinal cord lysates using NucleoMag B-beads (Macherey-Nagel). DNA concentration and purity were assessed using a DeNovix spectrophotometer and a Qubit fluorometer (Thermo Fisher). To assess the presence of Cre-mediated recombination, the *Cnr1* locus was amplified using primers P50 (5′-GCTGTCTCTGGTCCTCTTAAA-3′) and P53 (5′-CTCCTGTATGCCATAGCTCTT-3′) (Figure S1a). This reaction produced a ~ 2900 bp amplicon for the unrecombined allele and a ~ 689 bp product corresponding to the excised DNA fragment following Cre-mediated recombination. The presence of the floxed allele was evaluated in a separate reaction using primers P50 and P51 (5′-GGTGTCACCTCTGAAAACAGA-3′), which resulted in a ~ 595 bp product for the floxed allele and a ~ 475 bp product for the wild-type allele (Figure S1a). To determine the presence of Cre recombinase, samples were amplified with primers 5’-CGGCATGGTGCAAGTTGAATA-3’ and 5’-GCGATCGCTATTTTCCATGAG-3’, generating a band with 300 bp. PCR products were resolved by capillary electrophoresis and visualized using LabChip^®^ GX (Revvity).

### Quantitative RT-PCR

Mice were anesthetized with ketamine/xylazine (80/10 mg/Kg, i.p; Imalgene^®^/Rompun^®^) and transcardially perfused with cold PBS for 30–60 s with an average of an average of 5 mL buffer per mouse in order to remove circulating blood cells. The lumbar spinal cord was dissected in lysis buffer containing 1% β-mercaptoethanol for optimal template preservation. Total RNA was purified with on-column DNAse treatment using RNeasy Plus Mini kit (Qiagen; 74104) following manufacturer’s instructions. RNA was eluted with 14–35 µL of RNAse-free deionized water and stored at -80 °C until analysis. Synthesis of cDNA, pre-amplification and amplification steps were performed at the Genome Analysis Platform of the UPV/EHU following quality control of RNA samples with an Agilent 2100 Bioanalyzer (Agilent Technologies). Pre-amplified cDNA samples were measured with no reverse transcriptase and no template controls in the BioMark HD Real-Time PCR System using 48.48 Dynamic Arrays of integrated fluidic circuits (Fluidigm Corporation). We used commercial primers from IDT Integrated DNA Technologies or Fluidigm Corporation (Table S2). Data pre-processing and analysis were completed using Fluidigm Melting Curve Analysis Software and Real-time PCR Analysis Software (Fluidigm Corporation) to determine valid PCR reactions. *Gapdh*, *Hprt* and *Ppia* were included as candidate reference genes for normalization purposes. Data were corrected for differences in input RNA using the geometric mean of reference genes selected according to results from the normalization algorithms geNorm (https://genorm.cmgg.be/) and Normfinder (https://moma.dk/normfinder-software). Relative expression values were calculated with the 2^−ΔΔCt^ method.

### Western blot

Anesthetized mice were transcardially perfused with cold PBS and the lumbar spinal cords were homogenized (1:20 w/v) in ice-cold RIPA buffer (Thermo Fisher; #89900) containing a protease inhibitor cocktail (Thermo Fisher; #87786) using a Potter homogenizer with a loosely fitting Teflon pestle. Samples were incubated in ice for 30 min and subjected to centrifugation (12000 *x* g at 4 °C for 8 min) to remove insoluble material. Solubilized proteins were quantified in the supernatants using the BioRad Protein Assay Kit (Protein Assay Reagents; 5000-114-13-15). Protein samples (4 µg) were loaded into polyacrylamide Criterion TGX Precast (BioRad) gels before electrophoretic transfer onto Nitrocellulose membranes (Amersham™ Protran^®^ Western blotting membranes, pore size 0.2 μm). Membranes were blocked for 1 h in Tris-buffered saline (TBS; 50 mM Tris, 200 mM NaCl; pH 7.4) with 0.05% Tween-20, 5% BSA. Subsequently, membranes were incubated overnight at 4 °C with primary antibodies raised against myelin basic protein (MBP; 1:1000; BioLegend; #808401), Claudin 4 (CLN-4; 1:500; Thermo Fisher; #32-9400), Cadherin 5 (CDH-5; 1:500; R&D Systems; #AF1002), Intercellular Adhesion Molecule 1 (ICAM-1; 1:500; R&D Systems; #AF796), Podocalyxin (PODXL; 1:500; R&D Systems; #AF1556), Vascular cell adhesion protein 1 (VCAM-1; 1:1000; Abcam; #ab134047), zona occludens (ZO-1; 1:1000; Invitrogen; #02200) and α-Tubulin (1:5000; Abcam; #ab7291) in blocking solution. Membranes were incubated for 1 h at RT with horseradish peroxidase-conjugated secondary antibodies (1:5000; Cell Signaling Technology) and developed with NZY Standard ECL Western Blotting Substrate (NZYtech). Volumetric analysis of relevant immunoreactive bands was carried out after acquisition on a ChemiDoc XRS System (Bio-Rad) controlled by The Quantity One software v 4.6.3 (BioRad).

### Histology and fluorescence immunohistochemistry

#### Mice sacrifice and tissue processing

EAE mice were deeply anesthetized by intraperitoneal injection of Dolethal (200 mg/Kg) and perfused with 4% paraformaldehyde (PFA) in 0.1 M phosphate buffer (PB) (25 mM NaH_2_PO_4_·H_2_O, 75 mM Na_2_HPO_4_; pH 7.4) for 10 min. The spinal cords were extracted and post-fixed in the same fixative solution for 24 h at 4 °C. Alternatively, anesthetized mice were transcardially perfused with cold PBS and the brains extracted and stored at -80 °C. Mice injected with LPC were perfused with 2% PFA in 0.1 M PB for 15 min and spinal cords post-fixed for 30 min. Spinal cords were dissected into 1 or 4–5 longitudinal 2-mm-thick blocks containing either the demyelinating LPC lesion or equivalent lumbar spinal cord portions from EAE mice, respectively. Tissue blocks were then placed in 15% sucrose-7% gelatin in PBS, frozen in isopentane for 2 min at -65 °C and stored at -80 °C. Coronal 10–12 μm-thick spinal cord and forebrain sections were cut into Superfrost glass slides (Thermo Fisher, #11976299) using a CM3050 S cryostat (Leica Biosystems) and stored at -20 °C.

Mice injected with VEGF-A_165_ received a subcutaneous injection of buprenorphine (0.1 mg/kg) (Vetergesic) 30 min before surgery. Mice were then profoundly anesthetized by i.p. injection of a mix of ketamine (100 mg/kg; Imalgene) and xylazine (20 mg/kg; Rompun), and transcardially perfused with PBS for 5 min and then with 10% formalin (Merck; #252549) for 12 min. The brains were post-fixed in 10% formalin for 3 h and incubated in 30% sucrose overnight. Forebrain tissues were embedded on Tissue-Tek O.C.T. Compound (Sakura, #4583) and stored at -80 °C. Coronal 12 μm-thick sections containing the marked lesion were cryostat sectioned into Superfrost glass slides and stored at -20 °C until further processing.

#### Luxol fast blue myelin staining

Spinal cord sections were incubated overnight at 37–42 °C with 0.1% luxol fast blue (LFB) (Sigma-Aldrich; 1328-51-4) diluted in 95% ethanol and 0.5% glacial acetic acid and subsequently differentiated with a 0.01% lithium carbonate solution. Tissues were dehydrated by immersion in increasing concentrations of ethanol, processed with xylene and mounted with DPX mounting medium.

#### Hematoxylin and eosin staining

Spinal cord sections were rinsed in distilled water, incubated in hematoxylin solution (Epredia; #6765008) for 2 min and rinsed for 3 min in 0–3% clorhidric acid. Tissues were subsequently counterstained by incubation in eosin solution (Epredia; #6766008) for 25 min and rinsed in tap water for 5 min. Sections were dehydrated by immersion in increasing concentrations of ethanol, processed with xylene and mounted with DPX.

#### Immunolabelling of spinal cord and cortical tissue

Spinal cord sections were air-dried for 30 min at RT and rehydrated in TBS (20 mM Tris, 1.4 M NaCl; pH 7.6) for 30 min. Antigen retrieval was performed for OLIG2 immunostaining by adding low-pH R-Universal retrieval buffer (Aptum Biologics; #AP0530-500) and heating the slices in a microwave for 45 s at maximum temperature. For MBP immunolabelling, slices were permeabilized in absolute ethanol for 15 min at -20 °C followed by extensive washing in TBS. Tissue sections were incubated for 1 h at RT in blocking solution containing 5–10% normal goat serum (NGS) (Vector Labs; S-1000) or donkey serum (NDS) (Interchim; #UP77719A) and 0.2% Triton X-100 in TBS. The blocking solution was supplemented with 3% Fab fragment (Jackson ImmunoResearch) when using primary antibodies made in mouse. Slides were incubated overnight at 4 °C with primary antibodies (Table S3) diluted in TBS containing 5% NGS and 0.1% Triton X-100, washed in TBS (3 × 10 min) and incubated for 1 h at RT with Alexa Fluor secondary antibodies made in goat or donkey and Hoechst (4 µg/mL) (Sigma-Aldrich; B2261) in antibody solution. Tissue sections were washed with TBS (3 × 10 min) and mounted with ProLong Gold Antifade (Thermo Fisher; #P36930) or Fluoromount-G (Thermo Fisher; #00-4959-52) mounting media for microscopy analysis.

Cryostat sections from EAE mice containing the somatosensory cortex were air-dried for 30 min at RT, fixed in 4% PFA for 15 min and washed in TBS (3 × 10 min). Tissue slides were incubated for 1 h at RT in a blocking-permeabilization solution containing 5% NGS and 0.2% Triton X-100 in TBS and subsequently incubated for 12–48 h at 4 °C with primary antibodies (Table S3) diluted in blocking solution. Following extensive washing, primary antibodies were detected by incubation with appropriate Alexa Fluor secondary antibodies for 2 h at RT. Hoechst was used for chromatin staining. Sections were then washed in TBS (3 × 10 min) and mounted in ProLong mounting medium using coverslips for microscopy analysis.

Tissue sections from VEGF-A_165_-injected mice were tempered for 20 min and then rehydrated in PBS for 20 min. Antigen retrieval was performed by incubation in a Tris-EDTA solution (10 mM Tris HCl, 1 mM EDTA; pH 8) for 30 min at 100 °C. Slices were then washed in PBS (3 × 5 min) and incubated for 1 h at RT in a blocking solution containing 10% NDS and 0.3% Triton X-100 in PBS. Sections were subsequently incubated overnight at 4°C with the corresponding primary antibodies (Table S3) prepared in 5% NGS and 0.1% Triton X-100 in PBS. Tissue sections were washed in PBS (3 × 5 min) and incubated with Alexa Fluor secondary antibodies for 1 h at RT. Following extensive washing tissues were mounted on glass coverslips using Fluoromount-G with DAPI mounting medium (Thermofisher; #00-4959-52) for microscopy analysis. All the immunohistochemical experiments included tissue samples run in parallel without primary antibodies as internal controls.

#### Image acquisition and analysis

Quantitative analysis of demyelination and inflammatory lesion number in EAE mice was performed in 4–10 tissue sections per mouse imaged using a 3D Histech Panoramic MIDI II slide scanner and the CaseViewer and CaseConverter softwares (3DHistech). For the immunohistochemical characterization of brain and spinal cord tissues, optical images from sections processed in parallel were acquired using 20X and 40X lens on a Leica TCS STED CW SP8 super-resolution microscope or a Zeiss Axioplan 2 pseudoconfocal microscope coupled to an Axiocam MRc5 digital camera. Image acquisition was carried out using fluorescence intensity settings at which the control sections without primary antibody gave no signal.

Immunohistochemical characterization of demyelinating lesions in the EAE model was performed by examining 4 objective pictures taken from 2 non-consecutive spinal cord sections per mouse separated by ~ 2 mm. Regions of interest (ROIs) corresponding to the demyelinated plaque and the adjacent periplaque were established based on MBP immunohistochemistry and on the density of the cell nuclei. The plaque of demyelinated lesions was characterized by the total lack of MBP immunostaining and a high nuclear density, while the periplaque was determined as the area corresponding to a 100 μm perimeter measured from the lesion edge to the adjacent area, and characterized by weak or less dense MBP immunostaining. Immunolabelling of LPC injected spinal cords was evaluated in 2 tissue sections containing the central part of the demyelinating lesion. The somatosensory cortex of EAE mice was examined for inflammatory lesion load and astrocyte reactivity and immunohistochemical characterization performed in 2–4 images collected from 2 non-consecutive coronal sections per mouse separated by 150–200 μm. Cortical lesions induced by VEGF-A_165_ were evaluated in 3–4 images collected from the lesion area.

Image analysis was performed using Fiji Image J [45]. Immunopositive cells were counted in a selected ROI using a cell counter plugin and data expressed as mean cell number per square millimeter (mm^2^) of tissue area. Analysis of immunostained areas was performed in 16-bit gray scale transformed pictures. Fluorescence signals were considered positive if they were above a defined intensity threshold and normalized to total selected ROI area. For colocalization analysis, pixels positive for GFAP or aquaporin-4 and C3, ICAM-1, VCAM-1 or VEGF-A immunoreactivity were counted in projections of Z series stacks with the same number of images taken at a spacing of 0.8 μm by a blinded observer.

### Data collection and statistical analyses

No statistical methods were used to pre-determine sample sizes but they are similar to those reported in previous publications. Experimenters were always blinded to mice genotype but not to treatments. Statistical analyses were performed using GraphPad Prism 10 for Windows (GraphPad Software Inc). Summary results are presented as the mean of independent data points ± SEM. Individual datasets were initially tested for normal distribution with the Shapiro-Wilk test and differences between groups were determined by two-tailed unpaired Student *t* test, Mann-Whitney test or Wilcoxon matched-pairs signed rank test. Differences in EAE disease progression over time were assessed with the Wilcoxon matched-pairs signed-rank test. Calcium responses in the EAE model were analyzed using two-way ANOVA followed by Šídák’s test for multiple comparisons. Differences were considered to be significant when *p* < 0.05.

## Results

### Astrocyte CB_1_ receptors exacerbate clinical deficits and myelin pathology in EAE

**Fig. 1:**
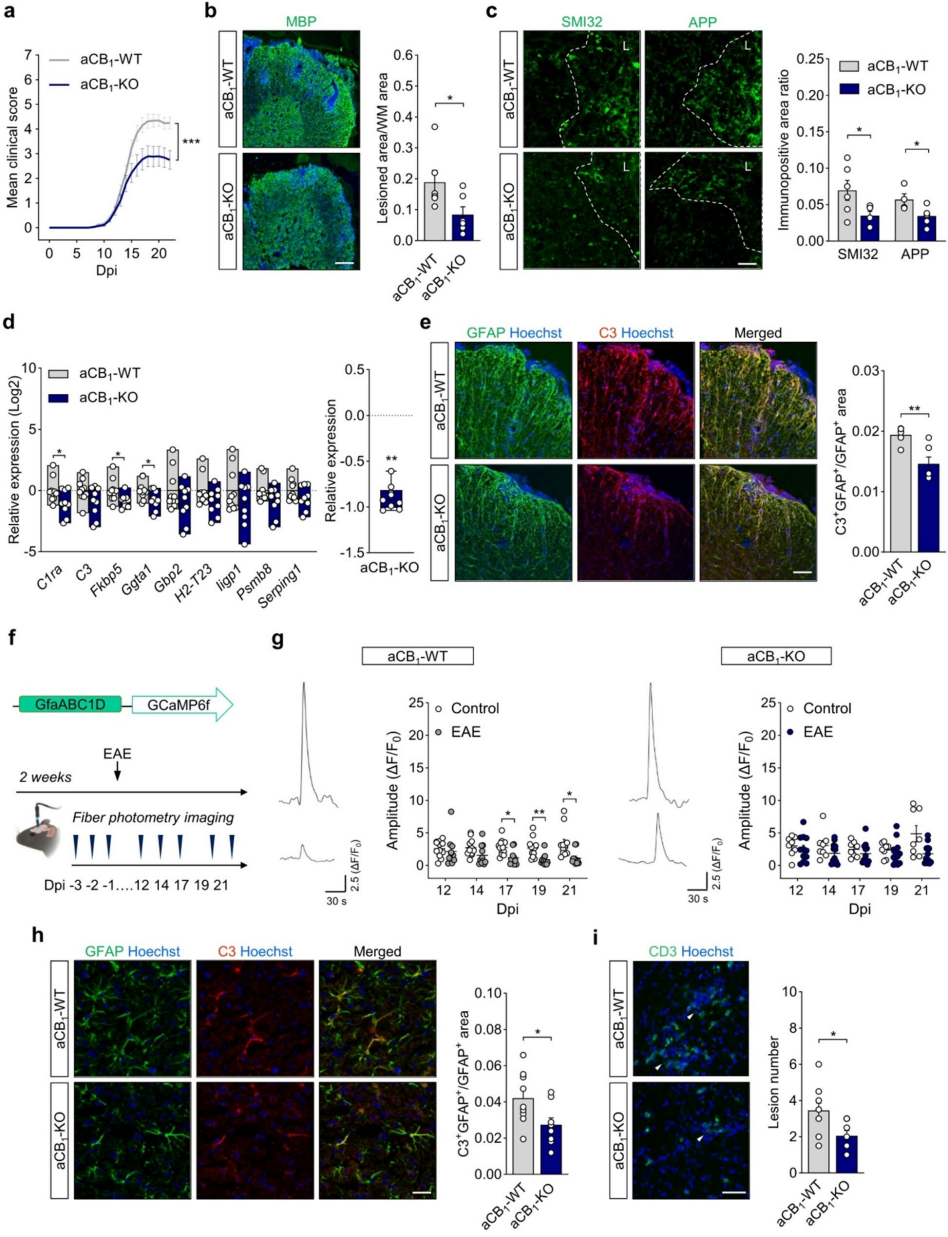
Astrocyte CB_1_R exacerbate EAE pathology and clinical deficits. **a** Time-course of EAE progression in aCB_1_-KO mice. Data are representative of 2 independent EAE experiments pooled together (*n* = 18–19 mice; ***p < 0.0001; Wilcoxon matched-pairs signed rank test for the comparison of score curves from the onset of EAE symptoms at 8 dpi to 22 dpi). **b**-**c** Representative images and/or quantitative analysis of MBP, SMI32 and APP immunostaining in spinal cord sections at 22 days post-immunization (dpi) (*n* = 6 mice). The dashed line delimits the demyelinating lesion (L) in **c**. Scale bar = 200 μm (**b**) and 25 μm (**c**). **d** Relative expression of neurotoxic astrocyte genes in spinal cord tissue (*n* = 9–10 mice). *Right panel*: Comparative analysis of neurotoxic astrocyte gene expression profile between genotypes (*n* = 9 genes) (*p* = 0.0039; Wilcoxon matched-pairs signed rank test). The expression of inflammatory genes in aCB_1_-KO mice was normalized to the mean expression of each gene in the control group. **e** Representative confocal z-stack projections and quantification of GFAP and complement component 3 (C3) colocalization in spinal cord tissue (*n* = 6 mice). Scale bar = 100 μm. **f**-**i** Attenuated cortical astrocyte pathology and functional deficits in aCB_1_-KO mice during EAE. **f** In vivo time-course analysis of astrocytic calcium activity for 3 consecutive days before EAE induction and at 12, 14, 17, 19 and 21 dpi. **g** Representative traces show calcium responses of cortical astrocytes evoked by tail-holding at -3 (*top*) and 19 (*bottom*) dpi. Graphs depict the amplitude of cortical astrocyte calcium responses recorded during the EAE time course compared to values from non-immunized animals recorded in parallel (*n* = 8–12 mice). **h** Confocal micrographs and quantification of C3 immunoreactivity in GFAP immunopositive astrocyte profiles within somatosensory cortex layer V-VI at acute EAE disease (*n* = 8–9 mice). Scale bar = 25 μm. (**i**) Confocal micrographs and quantification of CD3 immunopositive T inflammatory lesions (arrowheads) in cortical tissue (*n* = 8–9 mice). Scale bar = 40 μm. **b**-**e** and **h**-**i**, *p < 0.05, **p < 0.01 and ***p < 0.001, unpaired *t*-test or Mann-Whitney test. **g**, *p < 0.05 and **p < 0.01, two-way ANOVA followed by Šídák’s test for multiple comparisons. Error bars express SEM

To study the role of aCB_1_R in MS we analyzed the phenotype of conditional mutant mice lacking CB_1_R in GFAP positive cells [29, 32] in the EAE model of autoimmune demyelination. Upon EAE induction, mice lacking CB_1_R specifically in astrocytes (aCB_1_-KO) displayed similar disease onset but significantly decreased clinical scores during the acute phase of the disease (Fig. 1a) as well as at the experimental end-point (score at 22 dpi = 4.250 ± 0.240 in aCB_1_-WT *versus* 2.750 ± 0.377 in aCB_1_-KO; *n* = 18–19 mice; *p* = 0.0073; Mann-Whitney test). Histological evaluation of spinal cords at 22 dpi revealed that the number of inflammatory lesions (Figure S2a), predominantly found in white matter areas close to the tissue edge, and the proportion of demyelinated white matter (Fig. 1b), were reduced in aCB_1_-KO mice as compared to aCB_1_-WT controls. Consistent with a preserved neurological function, the levels of non-phosphorylated neurofilaments (SMI-32) and amyloid precursor protein (APP) within spinal cord lesions were significantly reduced in aCB_1_-KO mice, indicative of preserved neuroaxonal integrity (Fig. 1c). We next interrogated the protective phenotype of aCB_1_-KO mice at chronic EAE disease stages. Astrocyte-specific CB_1_R null mice displayed a sustained reduction in disability scores during EAE progression to a more chronic clinical plateau (Figure S3a, *left panel*). Comparative analysis at 35 dpi evidenced a non-significant attenuation of motor symptomatology in aCB_1_-KO mice (Figure S3a, *right panel*) that was associated with improved spinal cord myelin pathology in terms of demyelinating lesion numbers (Figure S3b) and proportion of demyelinated white matter area (Figure S3c-d). Inflammatory spinal cord lesions from aCB_1_-KO mice at the chronic stage also showed reductions in the extent of neuroaxonal degeneration that were encompassed by an attenuated presence of microglia/macrophages and reactive astrocytes, as determined by double immunolabelling for MBP and SMI32, Iba1 or GFAP (Figure S3c, e). Thus, aCB_1_R exacerbate autoimmune inflammation and associated clinical symptomatology during EAE progression.

### CB_1_ receptor deletion prevents astrocyte dysfunction during EAE

To study the role of CB_1_R in the phenotypic transformation of astroglial cells during EAE we assessed the expression of molecules related to the acquisition of astrocyte pathogenic properties [46–48] in spinal cord tissue. Astrocyte-specific CB_1_R null mice showed reduced expression levels of several genes associated with the conversion of these cells to disease-promoting phenotypes at acute EAE disease as determined by real-time qPCR analysis of spinal cord lysates (Fig. 1d). We next examined inflammatory spinal cord lesions for the presence of complement component 3 (C3) as marker of pathogenic astrocytes in MS and EAE [47–49]. Double immunofluorescence staining of GFAP and C3 indicated reduced expression of both proteins in demyelinating spinal cord lesions from aCB_1_-KO mice that were encompassed by lower numbers of Iba1^+^ microglia/macrophages (Figure S2b-c). Colocalization analysis of GFAP and C3 evidenced reductions in the expression levels of C3 within astrocytic profiles (Fig. 1e). These results may suggest that aCB_1_R facilitate the astrocyte transformation into pathogenic phenotypes during EAE.

We next sought for possible differences between genotypes regarding astrocyte functional properties. Reactive astrocytes in the somatosensory cortex display impaired calcium responses that correlate to the severity of clinical symptomatology during acute EAE [34, 49]. Thus, we reasoned that astrocyte-specific CB_1_R deletion might attenuate glial reactivity and preserve astrocyte network function in this brain area. Fiber photometry analysis of cortical astrocytes during EAE time-course (Fig. 1f) showed reductions in the amplitude of sensory-evoked calcium signals in freely behaving aCB_1_-WT mice at acute disease as compared to non-immunized mice (Fig. 1g, *left panel*). This result resembles our recent observations on astrocyte calcium deregulation in this rodent model of MS using non-transgenic mice [49] and adds to the growing body of evidence showing that attenuation of aCB_1_R function encompasses the pathogenic activation of these cells during acute autoimmune inflammation [34, 46]. Comparison of astrocyte calcium signals recorded from non-immunized aCB_1_-KO and aCB_1_-WT mice did not evidence differences between genotypes (Figure S4) showing that aCB_1_R deletion does not modulate calcium responses under our experimental paradigm in control conditions. However, astrocyte calcium signals evoked by sensory stimulation in the brain cortex of aCB_1_-KO mice at acute EAE disease were not significantly reduced as compared to those recorded in the control aCB_1_-KO group (Fig. 1g, *right panel*). Together, these combined observations suggest an attenuation of disease-associated astrocyte hypo-responsiveness at the calcium signaling level in mice lacking aCB_1_R. Consistently, cortical GFAP levels were reduced in the aCB_1_-KO group when compared to aCB_1_-WT mice without reaching statistical significance (*p* = 0.0806; unpaired *t* test) while C3 immunostaining was significantly downregulated (Figure S2d). Colocalization analysis showed astrocyte-specific reductions in C3 expression within deep cortical layers of aCB_1_-KO mice at acute disease (Fig. 1h). Furthermore, immunofluorescence staining revealed that the presence of cortical inflammatory lesions, defined by the presence of intraparenchymal CD3^+^ T cells, was also significantly reduced in aCB_1_-KO animals (Fig. 1i). Thus, aCB_1_R deletion attenuates cortical inflammation and astrocyte network dysfunction during EAE.

### Mice with astrocytic CB_1_ receptor inactivation display intact oligodendrocyte populations in the EAE and LPC models

**Fig. 2:**
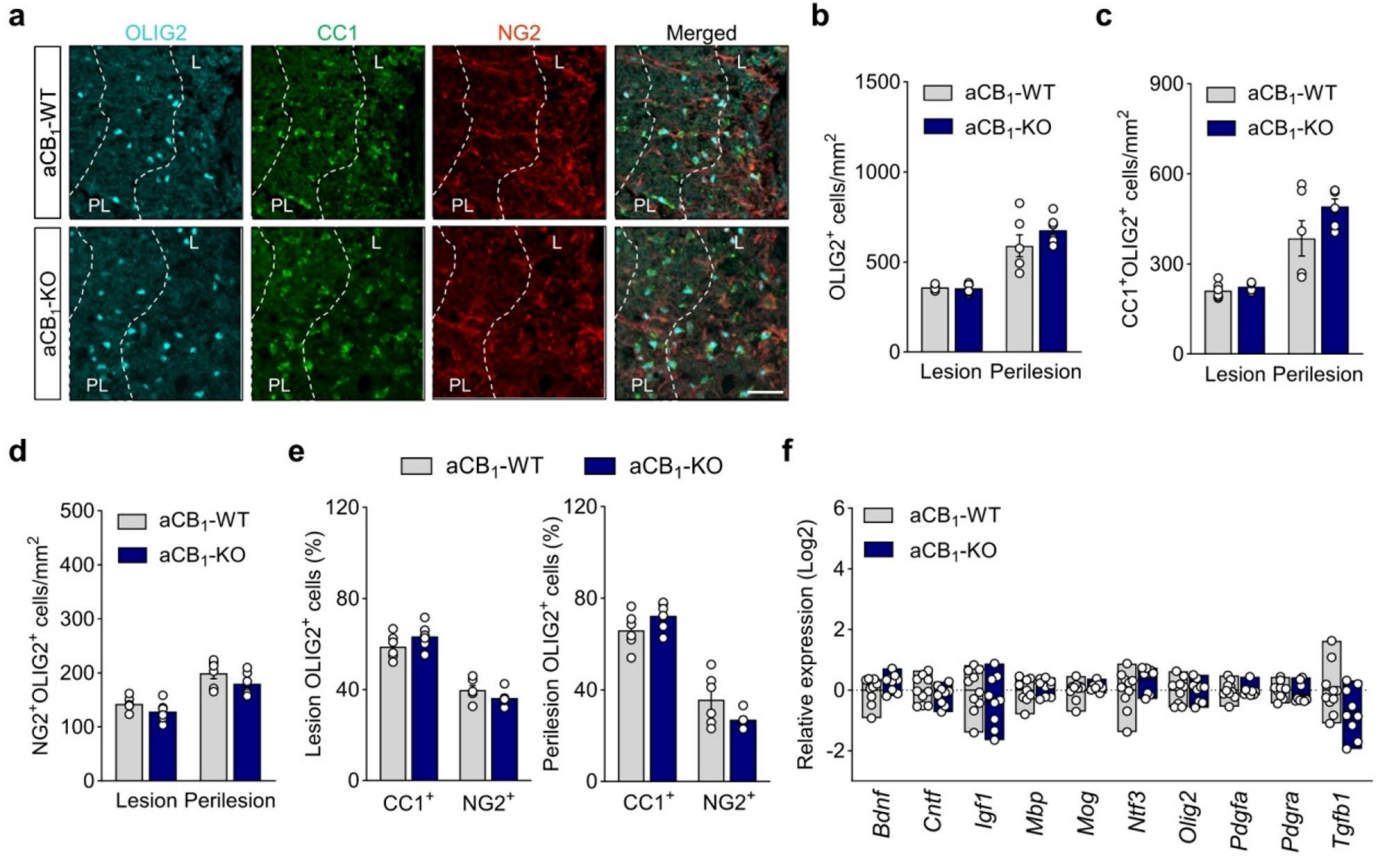
Astrocyte CB_1_R do not modulate oligodendrocyte populations in EAE inflammatory lesions. **a** Representative images of spinal cord lesions from aCB_1_-KO and aCB_1_-WT mice at acute EAE immunostained for OLIG2, CC1 and NG2 as markers of oligodendrocyte lineage cells, mature oligodendrocytes and oligodendrocyte precursor cells, respectively, show low cell densities in the lesion (L) and enrichment in the perilesion area (PL, dashed lines). Scale bar = 40 μm. **b**-**d** Quantitative analyses show similar densities of oligodendrocyte populations in the plaque and periplaque of aCB_1_-KO and aCB_1_-WT mice (*n* = 6 mice). **e** Percentages of oligodendrocytes (CC1^+^) and OPCs (NG2^+^) in the OLIG2^+^ population indicate equal proportions of mature cells in the lesion and perilesion of aCB_1_-KO and control littermates. **f** Gene expression analysis of oligodendrocyte/myelin markers and pro-myelinating molecules in spinal cord tissue from aCB_1_-KO and aCB_1_-WT mice at acute disease (*n* = 9–10 mice). Error bars express SEM

Oligodendrocyte differentiation prevents axonal loss and attenuates clinical symptomatology in the EAE model [50–52], thus pinpointing to functional remyelination as potential mechanism of disease attenuation during autoimmune inflammation. Neurotoxic astrocytes release factors that promote oligodendrocyte apoptosis and delay lineage progression, leading to reduced remyelination and subsequent neuronal death [8, 48]. Based on these evidences, we hypothesized that the protective phenotype of aCB_1_-KO mice in terms of astrocyte reactivity and demyelination extent may be related to the engagement of repair mechanisms during disease time-course. To determine whether aCB_1_R impede oligodendrocyte differentiation-promoting effects of astroglial cells as mechanism of clinical exacerbation, we immunostained spinal cord sections from aCB_1_-WT and aCB_1_-KO mice for the oligodendrocyte lineage marker OLIG2 in combination with CC1 and NG2 to identify myelinating oligodendrocytes and OPCs, respectively. Astrocyte-specific CB_1_R mutants at acute EAE disease displayed unaltered numbers of OLIG2^+^ oligodendrocyte lineage cells, CC1^+^/OLIG2^+^ oligodendrocytes and NG2^+^/OLIG2^+^ OPCs as compared to littermate controls, both in the demyelinating lesions and in the surrounding perilesion areas (Fig. 2a-d). The percentages of CC1^+^/OLIG2^+^ mature oligodendrocytes and NG2^+^/OLIG2^+^ OPCs in the plaques and periplaques were also similar between aCB_1_-WT and aCB_1_-KO mice (Fig. 2e). Thus, aCB_1_R do not modulate oligodendrocyte populations at acute EAE disease. Consistently, gene expression analysis of oligodendrocyte/myelin genes (*Olig2*, *Pdgfra*,* Mbp*, *Mog*) and factors that promote oligodendrocyte differentiation and (re)myelination (*Bdnf*, *Cntf*,*Ifg1*, *Ntf3*, *Pdgfa*, *Tgfb1*) did not show significant differences between genotypes (Fig. 2f). Collectively, these results suggest that aCB_1_R do not hinder myelin repair as mechanism of clinical deterioration during EAE.

Mechanistic studies of remyelination in EAE mice are challenging as autoimmune inflammation produces concomitant demyelination, axonal damage and myelin repair [53]. To gain further insights on the role of aCB_1_R during remyelination in vivo we used a toxin-induced model in which demyelination of focal lesions generated by localized injection of LPC in spinal cord white matter is followed by spontaneous remyelination [54–56]. OPCs are recruited into the demyelinated lesion between 3 and 7 days post-lesion (dpl) and differentiate to mature oligodendrocytes during the second week post-lesion, thus providing a defined time window to study changes in the rate of remyelination. We analyzed oligodendrocyte populations in LPC lesions from aCB_1_-WT and aCB_1_-KO mice at 14 dpl corresponding to the peak of endogenous OPC differentiation during the remyelination phase (Figure S5a). The demyelination extent of LPC lesions, assessed by co-immunostaining against MBP, was similar between genotypes (Figure S5b). Quantification of OLIG2^+^ oligodendrocyte lineage cells and double-labelled CC1^+^/OLIG2^+^ mature and CC1^−^/OLIG2^+^ immature populations in LPC lesions revealed no variations between aCB_1_-KO and aCB_1_-WT mice (Figure S5c-e). Consistently, the numbers of PDGFRα^+^ precursor cells in LPC lesions were similar in aCB_1_-KO and aCB_1_-WT mice (Figure S5f). We also investigated potential changes in oligodendrocytes expressing brain-enriched myelin-associated protein 1 (BCAS1), which have been identified as a population of immature cells actively involved in (re)myelination [57, 58]. Immunohistochemistry for BCAS1^+^ revealed significant numbers of active oligodendrocytes in the lesion area of both aCB_1_-KO and aCB_1_-WT mice that remained unaltered between genotypes (Figure S5g). Thus, aCB_1_R do not modulate spontaneous OPC differentiation following toxic oligodendrocyte loss. We next assessed LPC lesions from aCB_1_-KO and aCB_1_-WT mice for the presence of inflammatory cells. GFAP expression was not significantly different between groups, suggesting that LPC injections activate astrogliosis to a similar extent in aCB_1_-KO and aCB_1_-WT mice (Figure S5h). The presence of Iba1^+^ microglia/macrophages (Figure S5i) and CD45 infiltrating inflammatory cells (Figure S5j) was also similar between genotypes. These combined results suggest that aCB_1_R do not interfere with myelin repair processes in inflammatory and toxin induced demyelinating contexts and point to alternative mechanisms for clinical exacerbation during autoimmune inflammation.

### Reduced humoral and cellular infiltration in EAE lesions from astrocyte-specific CB_1_ receptor null mice

**Fig. 3:**
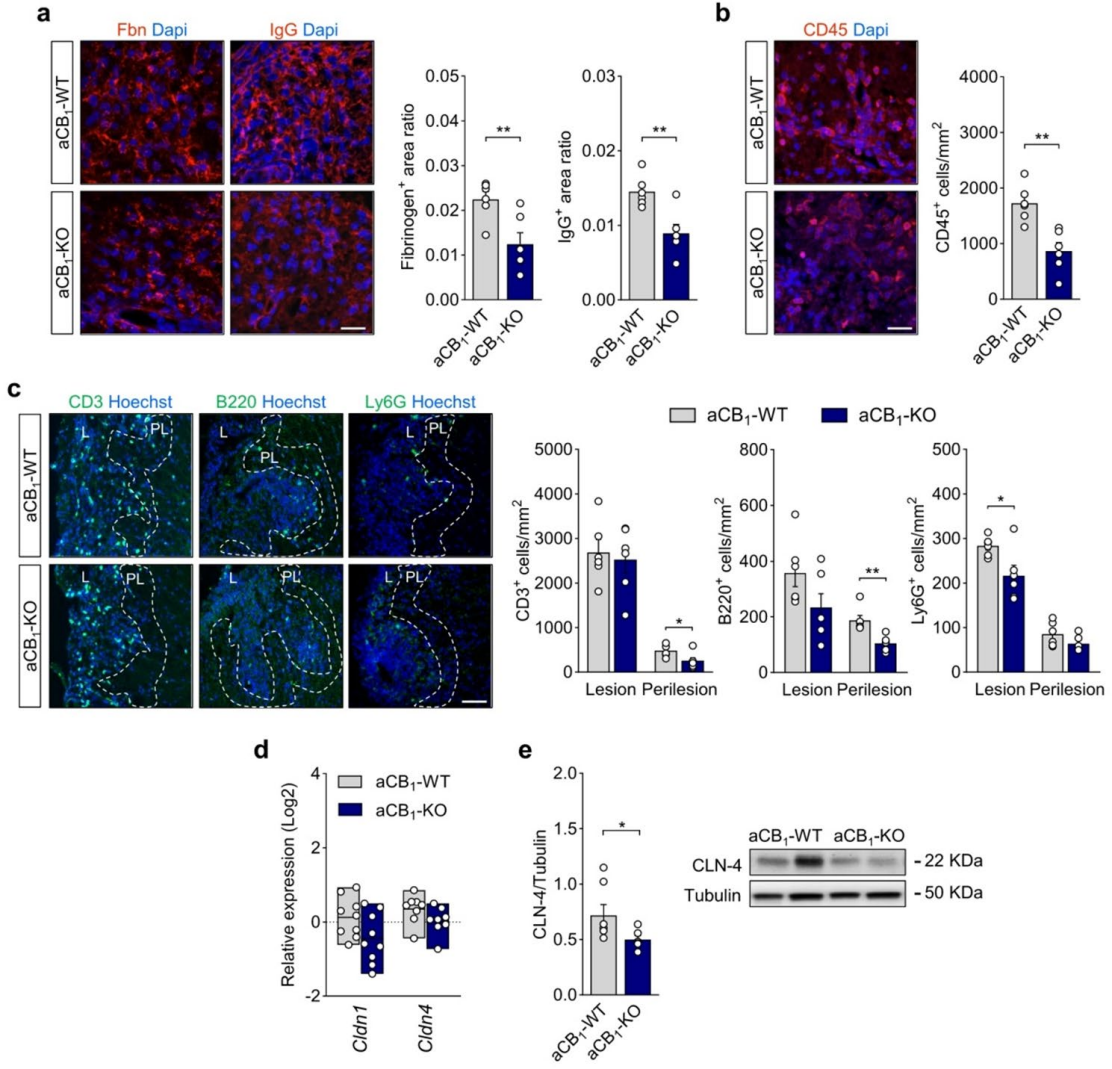
Restricted humoral and immune cell infiltration in EAE inflammatory lesions of aCB_1_-KO mice. **a** Representative confocal z-stack projections of spinal cord lesions from aCB_1_-KO and control aCB_1_-WT mice at acute EAE (25 dpi) immunostained for fibrinogen (Fbn) and IgG. Morphometry of parenchymal immunoreactivity shows reduced protein extravasation, measured as immunopositive area normalized to demyelinating lesion area, in aCB_1_-KO mice compared to the aCB_1_-WT group (*n* = 6 mice). Scale bar = 25 μm. **b** Representative micrographs and quantification of CD45^+^ infiltrating inflammatory cells in demyelinating lesions of aCB_1_-KO and aCB_1_-WT mice (*n* = 6 mice). Scale bar = 25 μm. **c** Confocal images and quantitative analysis of CD3^+^ T cells, B220^+^ B cells and Ly6G^+^ neutrophils in lesion (L) and perilesion (PL, dashed lines) spinal cord tissue from aCB_1_-KO mice as compared to aCB_1_-WT animals at acute EAE disease (*n* = 6 mice). Scale bar = 50 μm. **d** Gene expression analysis of the astrocyte TJ proteins CLN-1 and CLN-4 in spinal cord tissue from aCB_1_-KO and aCB_1_-WT mice at 22 dpi (*n* = 9–10 mice). **e** Immunoblots and densitometry analysis of CLN-4 expression in spinal cord lysates from aCB_1_-KO at 25 dpi compared to the aCB_1_-WT group (*n* = 6–7 mice). *p < 0.05, **p < 0.01 and ***p < 0.001, unpaired *t*-test or Mann-Whitney test. Error bars express SEM

Perivascular astrocyte processes are enriched in CB_1_R that modulate BBB permeability during stress-induced inflammation [59–62]. Thus, we wondered whether changes in the BBB properties contribute to the protective phenotype of astrocyte-specific CB_1_R null mice during EAE. To address this question, we initially measured parenchymal entry of humoral factors and immune cells as readouts of BBB opening at acute EAE disease [40, 63, 64]. The areas immunopositive for the serum proteins fibrinogen and IgG as markers of humoral factor extravasation were markedly reduced in spinal cord lesions from aCB_1_-KO mice (Fig. 3a). Moreover, spinal cord tissues from aCB_1_-KO mice contained significantly lower numbers of CD45^+^ leukocytes (Fig. 3b) and reduced densities of CD3^+^, B220^+^ and Ly6G^+^ leukocyte subsets (Fig. 3c) within EAE lesions or perilesion areas, as measured using histopathology. Of note, the comparative analysis of splenic lymphoid and myeloid cell populations revealed no detectable differences between non-immunized aCB_1_-WT and aCB_1_-KO mice in any of the evaluated cellular subsets (Figure S6). This result suggest that astrocyte-specific CB_1_R mutants do not display an impaired ability to develop autoimmune responses, consistent with the lack of differences between genotypes in terms of symptom onset. Collectively, these observations highlight that aCB_1_-KO mice display preserved BBB function during autoimmune inflammation.

Astrocytes promote the formation of demyelinating lesions in EAE and MS by mechanisms that lead to the repression of endothelial tight junction (TJ) proteins and drive BBB permeability [63, 64]. Concomitantly, astroglial cells activated in response to neuroinflammation upregulate claudin 1 (CLN-1) and claudin 4 (CLN-4) to form protective astrocytic TJ complexes that control humoral and cellular transit [65]. Expression of *Cldn1* and *Cldn4* in spinal cord tissue was not significantly modulated in the aCB_1_-KO group as compared to aCB_1_-WT mice (Fig. 3d). However, western blot analysis of spinal cord lysates showed reduced expression levels of the main astrocytic TJ protein CLN-4 [65] (Fig. 3e), which sits well with the attenuated inflammatory phenotype of aCB_1_-KO mice (Fig. 3b-c). To investigate whether aCB_1_R modulate the endothelial BBB during autoimmune inflammation we analyzed the expression of vascular endothelial cell markers and TJ associated proteins in spinal cord tissues from EAE mice. Astrocyte-specific CB_1_R null mice displayed unaltered levels of the vascular endothelial membrane proteins podocalyxin (PODXL) and laminin, as measured using immunoblotting and confirmed by immunohistochemistry of EAE lesions (Figure S7a-b). These findings indicate that aCB_1_-KO mice do not display abnormal angiogenesis at acute EAE disease as compared to wild-type animals. The expression of the endothelial TJ components cadherin 5 (CDH-5), zonula occludens (ZO-1) and claudin 5 (CLN-5) was also similar between aCB_1_-WT and aCB_1_-KO mice (Figure S7c-d). Thus, aCB_1_R null mice display microvascular endothelial cells similar to wild-type controls at established EAE disease despite the attenuated humoral and leukocyte infiltration in lesion and perilesion sites.

### Mice with astrocyte CB_1_ inactivation show reduced expression of immune and vascular effector molecules in EAE lesions

**Fig. 4:**
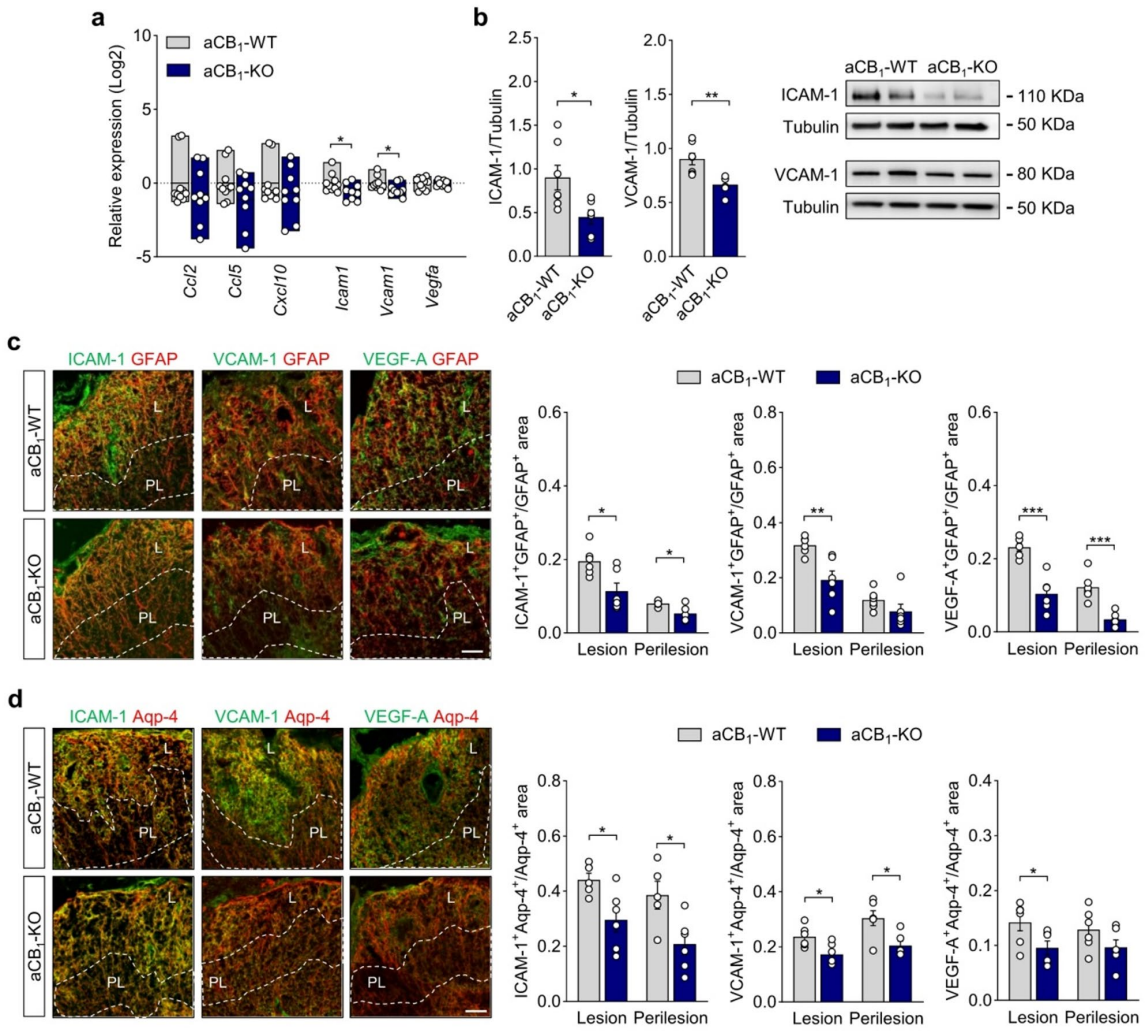
Astrocyte-specific CB_1_R mutants show reduced expression of immune and vascular effector molecules in EAE lesions. **a** Relative gene expression of permeability effector molecules in spinal cord tissue from aCB_1_-KO mice as compared to control aCB_1_-WT animals at acute disease EAE (*n* = 9–10 mice). **b** Immunoblotting for the adhesion molecules ICAM-1 and VCAM-1 shows reduced expression levels in aCB_1_-KO spinal cords (*n* = 6–7 mice). **c**-**d** Confocal z-stack projections of spinal cord sections double immunostained for (**c**) GFAP or (**d**) aquaporin-4 (Aqp-4) and ICAM-1, VCAM-1 or VEGF-A. Colocalization analysis highlights attenuated expression of permeability molecules in astroglial profiles within lesion (L) and/or perilesion (PL, dashed lines) areas from aCB_1_-KO animals (*n* = 5–6 mice). Scale bar = 50 μm. *p < 0.05, **p < 0.01 and ***p < 0.001, unpaired *t*-test or Mann-Whitney test. Error bars express SEM

Reactive astrocytes contribute to the pathogenesis of CNS inflammatory lesions through the release of intercellular molecules that enable circulating inflammation to reach the brain and spinal cord parenchyma. Astrocyte effector molecules that mediate autoimmune inflammation include the chemokines CCL2, CCL5 and CXCL2 as recruiters of perivascular leukocytes [8, 10], adhesion molecules such as ICAM-1 and VCAM-1 aberrantly expressed by astroglial cells [66–69], and angiogenic factors, mainly VEGF-A, that signal to the vascular endothelium and promote permeability [40, 63, 64]. To gain further insights on the mechanistic implications of aCB_1_R during autoimmune inflammation we addressed the expression of immune and vascular effector molecules at acute EAE disease. The expression levels of *Ccl2*, *Ccl5* and *Cxcl2* determined by RT-qPCR in spinal cord lysates of EAE mice were highly heterogeneous and not significantly modulated in the aCB_1_-KO group (Fig. 4a). Conversely, gene expression analysis highlighted significantly lower levels of *Icam1* and *Vcam1* that were confirmed by immunoblotting (Fig. 4a-b) and confocal imaging (Figure S8) of spinal cord tissue. Moreover, double immunostaining for GFAP or aquaporin-4 with ICAM-1 or VCAM-1 showed reduced expression of both adhesion molecules in astrocytic profiles within EAE lesions and perilesion areas of aCB_1_-KO mice (Fig. 4c-d). Of note, VEGF-A expression was drastically downregulated in spinal cord tissue (Figure S8) and GFAP and aquaporin-4 immunopositive astrocyte profiles (Fig. 4c-d) of aCB_1_-KO mice as compared to the aCB_1_-WT group. Thus, the inhibition of humoral and lymphocyte infiltration in white matter lesions of mice with aCB_1_R deletion is accompanied by a restricted astrocyte production of adhesion molecules and VEGF-A during autoimmune inflammation.

### Diminished inflammatory neuropathology of astrocyte CB_1_ receptor null mice in the VEGF-A model of BBB breakdown

**Fig. 5:**
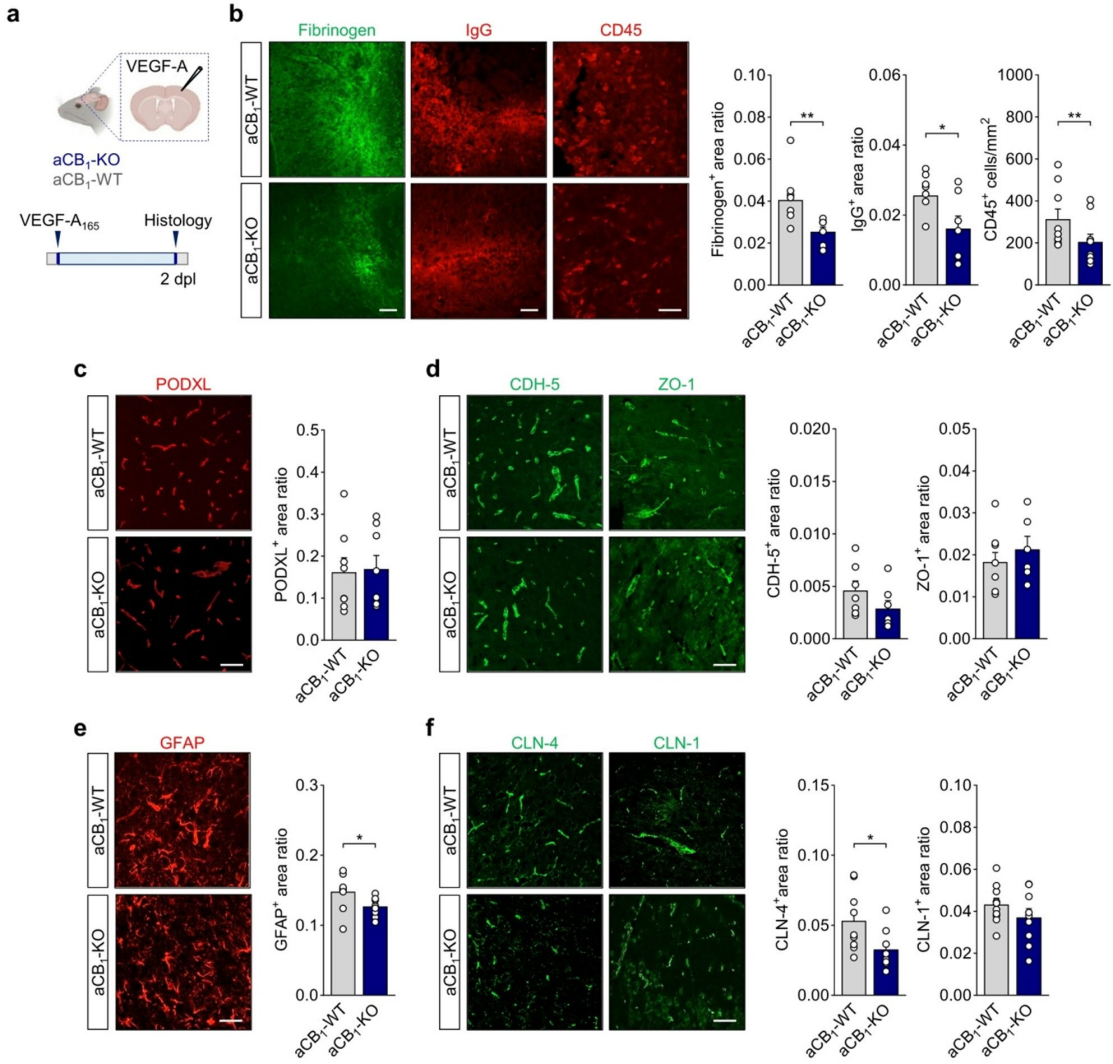
Astrocyte CB_1_R deletion reduces cortical BBB leakage, astrocytic tight-junction deregulation and leukocyte infiltration by VEGF-A. **a** Experimental design for the analysis of the BBB breakdown following cortical microinjection of murine VEGF-A_165_ in aCB_1_-KO and aCB_1_-WT mice. Cerebral cortices from animals injected in 2 independent experiments were processed for histopathology at 2 days post-injection (dpi). **b** Representative cortical tissue sections immunostained for fibrinogen, IgG and CD45 and quantitative analysis show reduced extravasation of serum proteins, measured as immunopositive area normalized to total lesion area, and attenuated leukocyte infiltration in the aCB_1_-KO group (*n* = 9 mice). Scale bars = 100 μm (fibrinogen, IgG) and 50 μm (CD45). **c**, **d** Confocal micrographs and morphometry of (**c**) PODXL and (**d**) endothelial TJ proteins CDH-5 and ZO-1 in aCB_1_-KO and aCB_1_-WT mice. Scale bar = 50 μm. **e**, **f** Attenuated astrocyte reactivity measured by immunostaining for GFAP (**e**) and levels of astrocyte TJ proteins CLN-4 and CLN-4 (**f**) following microinjection of VEGF-A_165_ in aCB_1_-KO and aCB_1_-WT mice. Scale bar = 50 μm. *p < 0.05 and **p < 0.01, unpaired *t*-test or Mann-Whitney test. Error bars express SEM

Collectively, the above findings suggest that aCB_1_R facilitate BBB permeability to promote inflammatory lesion formation during autoimmune inflammation. To further investigate potential links between aCB_1_R and BBB opening in vivo, we addressed the phenotype of astrocyte-specific CB_1_R null mice in the VEGF-A injection model, which triggers a rapid, localized breakdown of BBB structure and function mirroring pathological leakage during autoimmune inflammation [40, 63, 64]. We stereotaxically delivered mouse VEGF-A_165_ into the left cerebral cortex of aCB_1_-KO mice and littermate aCB_1_-WT animals and measured changes in barrier function at 2 days post-injection (Fig. 5a). Compatible with published data [40, 64], we observed BBB breakdown as measured by fibrinogen and IgG immunoreactivity in VEGF-A_165_-injected areas (Fig. 5b). The endothelial TJ markers CDH-5 and ZO-1 appeared patchy and discontinuous in both aCB_1_-WT and aCB_1_-KO mice (Fig. 5d) and these changes were accompanied by parenchymal accumulation of CD45^+^ cells (Fig. 5b). Importantly, BBB disruption, as measured by serum protein and immune cell extravasation, was attenuated in aCB_1_-KO mice (Fig. 5b) despite unaltered expression levels of the endothelial cell marker PODXL (Fig. 5c), and TJ proteins CDH-5 and ZO-1 (Fig. 5d). Reminiscent of our observations in the EAE lesions, aCB_1_R null mice showed reduced astrocyte reactivity (Fig. 5e), and lower levels of the astrocyte TJ protein CLN-4, but not of CLN-1, in areas of BBB disruption (Fig. 5f). These studies show that aCB_1_R engage mechanisms downstream of VEGF-A that promote BBB leakiness and allow for the infiltration of peripheral immune cells during inflammatory lesion formation.

## Discussion

Studies conducted during the past decades have demonstrated that CB_1_R signaling restricts clinical disability in MS [16, 21]. Potential mechanisms for (endo)cannabinoid-mediated protection mediated by CB_1_R in MS patients include neuroprotective and remyelination promoting effects, as defined by analyzing the phenotype of constitutive CB_1_R knockout mice [22] and transgenic mice lacking CB_1_R on neurons [20] and OPCs [25] in preclinical disease models. Nevertheless, the specific roles of CB_1_R expressed by astrocytes in MS have been scarcely investigated despite the critical involvement of these cells in disease initiation and progression [8–10]. This study is the first to our knowledge that highlights the significance of CB_1_R endocannabinoid signaling mediated by astrocytes in the generation of clinical deficits during CNS inflammatory disease. Upon characterizing the phenotype of mice lacking aCB_1_R in models of acute and chronic CNS damage, we depict an unprecedented deleterious role of CB_1_R in astroglial cells during inflammatory lesion formation linked to the modulation of BBB permeability. On mechanistic grounds, we show that aCB_1_R deletion restricts humoral and cellular leakage associated with BBB disruption, at least in part, by engaging cellular processes downstream VEGF-A signaling. These observations uncover a novel mechanism underlying astrocyte-related control of the BBB in neuroinflammation and shed light on the roles of endocannabinoids and CB_1_R in the pathogenesis of MS.

In this study, we show protective effects of aCB_1_R inactivation during the time-course of EAE at the clinical and neuropathological level. Attenuation of neurological disability occurred in concert with changes in astrocyte reactivity and expression of molecules related to the acquisition of disease promoting functions in MS patients and preclinical disease models [46, 48]. These observations are consistent with a scenario in which aCB_1_R signaling facilitates the emergence of neurotoxic phenotypes that impede astrocyte-oligodendrocyte interactions underlying myelin repair as potential mechanism of disease exacerbation [8]. Studies specifically designed to investigate the involvement of cell autonomous astrocyte responses mediated by CB_1_R in remyelination are currently lacking. Thus, we addressed this possibility by histologically targeting oligodendrocyte populations in lesion and perilesion areas of aCB_1_-KO mice at acute EAE disease. This analysis did not evidence significant modulatory effects encompassing the attenuation of inflammation and myelin pathology that resulted from deletion of CB_1_R in astrocytes. Consistently, analysis of remyelinating spinal cord lesions induced by microinjection of LPC did not highlight oligodendrocyte differentiation promoting effects in astrocyte-specific CB_1_R null mice. Although the possibility that endocannabinoids target CB_1_R in astroglial cells to modulate remyelination in vivo cannot be fully disregarded in the absence of a detailed myelin ultrastructure analysis during the time-course of myelin repair, these results suggest that aCB_1_R do not impede remyelination as potential mechanism of clinical exacerbation in demyelinating disease.

Our present data support the hypothesis that astrocyte responses to CB_1_R signaling modulate the formation of CNS lesions as principal mechanism of disease exacerbation. This possibility sits well with the fundamental roles of astroglial cells in regulating the BBB breakdown during CNS inflammation [8, 10] and with reports of enriched CB_1_R expression in perivascular astrocytic profiles [59, 61, 62]. Indeed, the clinical benefits of astrocyte-specific CB_1_R inactivation during EAE are observed during the onset of neurological symptomatology and occur in concert with reduced inflammatory lesion load at established disease, both in cortical and spinal cord tissue. These observations prompted us to investigate the phenotype of aCB_1_-KO mice in terms of BBB disruption during EAE. Here, as expected if CB_1_R in astrocytes would promote BBB permeability defects allowing circulating inflammation to enter the CNS parenchyma, aCB_1_-KO mice exhibited lower levels of humoral mediators and infiltrating leukocytes in inflammatory demyelinating EAE lesions and surrounding perilesion areas. Notably, these changes were associated with reduced expression levels of the adhesion molecules ICAM-1 and VCAM-1 by reactive astrocytes in EAE lesions. These findings may explain, at least in part, the restricted presence of infiltrating immune cells observed in mice with astrocyte-specific CB_1_R deletion, according to the established roles of both adhesion molecules in mediating leukocyte movement across the BBB during CNS inflammatory disease [66–69]. Reactive astrocytes in EAE lesions from aCB_1_-KO mice also exhibited lower levels of the angiogenic and pro-permeability factor VEGF-A, identified as a key driver of astrocyte-mediated BBB disruption, leukocyte infiltration and neuropathology during CNS inflammation [63, 64]. Reductions in astrocyte VEGF-A expression might limit leukocyte entry and contribute to reducing lesion load in astrocyte-specific CB_1_R null mice. Concomitantly, lower levels of adhesion molecules or other potential astrocyte mediators may further restrict BBB permeability as evolving inflammation drives astrocyte VEGF-A expression [70]. Using the focal, directly induced VEGF-A model in transgenic mice allowed us to selectively examine the role of CB_1_R-related astrocyte responses on BBB breakdown and lymphocyte entry downstream this vascular effect molecule. Critically, mice with astrocyte-specific CB_1_R deletion showed milder humoral and cellular extravasation induced by VEGF-A, thus mirroring the restricted inflammatory phenotype observed in EAE lesions. Altogether, these findings suggest that CB_1_R signaling in astrocytes facilitates the formation of CNS inflammatory lesions by targeting effector mechanisms downstream VEGF-A that promote BBB disruption.

Notably, the above changes occurred in concert with reduced levels of the astrocyte TJ protein CLN-4, whose induction has been associated with the emergence of a protective astrocyte barrier that limits the access of peripheral inflammation to the CNS parenchyma [65]. These seemingly contradictory findings can be explained on the basis that the evolving inflammation in lesion sites, which we find to be significantly reduced in mice lacking aCB_1_R, is a driving force for the induction of junctional adhesion proteins at the glia limitants [65]. A striking observation in this study, however, is that attenuated parenchymal leakage of circulating inflammation in lesions from aCB_1_-KO mice was not encompassed by significant endothelium abnormalities, as expected according to reports that BBB breakdown results from disruptions of endothelial junctional proteins such as CLN-5 [40, 63, 64]. It should be borne in mind, however, that our present findings do not exclude the possibility that astrocyte-specific CB_1_R null mice exhibit preserved endothelial barrier functions at initial stages of BBB dysfunction in the EAE and VEGF-A models, as compared to non-transgenic animals. Disruption of the BBB precedes immune cell infiltration during CNS lesion formation [40, 71] and it seems plausible that early-onset differences in endothelial permeability between genotypes are masked at later states by the evolving inflammatory neuropathology. Future research targeting the time-course of CNS lesion formation in vivo combined with clinically relevant in vitro models of BBB dysfunction that combine pharmacological approaches and gene knockdown strategies may uncover the mechanistic basis of astrocyte signaling to endothelial cells under the control of endocannabinoids and aCB_1_R that lead to clinical exacerbation during demyelinating disease. In this context, the paucity of studies that investigate the signaling events engaged by endocannabinoids at the BBB permeability level [60, 72, 73] contrasts the overwhelming evidence supporting that these lipid mediators and their exogenous counterparts dampen CNS inflammation in multiple disease paradigms [14, 16]. Result from the Theiler’s murine encephalomyelitis virus (TMEV) infection model supported a protective role for CB_1_R expressed by endothelial cells in attenuating leukocyte transmigration [74]. More recently, perivascular aCB_1_R have been postulated to promote stress resilience in mice through the preservation of BBB functions [60]. In this landmark study, viral-mediated astrocyte overexpression of CB_1_R mitigated inflammatory responses and morphological changes in the chronic social defeat stress (CSDS) mouse model of depression. These observations are in apparent contrast with our present findings that aCB_1_R deletion preserves BBB function and restrains circulating inflammation to enter the brain parenchyma. It should be noted, however, that stress-induced inflammation and autoimmune demyelination are intrinsically different disease contexts that involve dissimilar etiopathological mechanisms. Furthermore, the protective phenotype of aCB_1_R at the vascular level reported by Dudek and collaborators was ascribed to astrocyte populations within the nucleus accumbens shell in male mice resilient to CSDS. Conversely, this study, that we performed in female mice, suggests more generalized mechanisms underlying the control of BBB permeability by aCB_1_R during CNS lesion formation, as aCB_1_-KO mice showed attenuated spinal cord white matter and cortical grey matter inflammatory pathology in complementary disease models. Remarkably, CSDS and viral-induced downregulation of endothelial *Cln5* increase aCB_1_R and endocannabinoid levels in vivo while acute inflammatory challenges with IL-6 upregulate the expression levels of *Cnr1* in culture systems [60]. These recent observations suggest that BBB disruption facilitates aCB_1_R signaling through inflammation-related mechanisms. In this scenario, it seems plausible that distinct inflammatory environments engage endocannabinoid signaling through specific astrocytic pools of CB_1_R located at plasmatic membranes and/or mitochondrial compartments, leading to context-specific modulation of BBB functions. Unfortunately, astrocyte-specific adaptations in CB_1_R function and signaling in the EAE model have not been investigated until recently. Gene expression analysis of endocannabinoid signaling genes in astrocytes purified during the time-course of EAE highlighted reduced *Cnr1* transcript levels both at acute (14–17 dpi) and chronic (28–31 dpi) stages that were absent at presymptomatic disease (7–8 dpi) [46]. More recently, ex vivo and in vivo analysis of astrocyte calcium dynamics in mice at acute EAE demonstrated reductions in CB_1_R coupling to the modulation of intracellular calcium responses that involve deficits in the intracellular pathways operated by G proteins [34]. In this study, impairments in aCB_1_R-mediated calcium signaling translated into gliotransmission defects leading to exacerbated synaptic excitation, with still unknown consequences in terms of cortical pathology. Given that aCB_1_R signaling is downregulated at acute EAE, we assume that the pathogenic activation of the astrocytic receptor pools in this rodent model autoimmune demyelination takes place during the initial phase of the disease, consistent with an early role in the formation of inflammatory lesions. Of note, astrocyte activation during EAE involves early-onset reductions in most endocannabinoid hydrolysis genes that can be detected at presymptomatic stages [46]. These findings provide a plausible cell autonomous mechanism for the early activation of aCB_1_R in this model of autoimmune inflammation potentially leading to receptor downregulation at later stages. Further, locally produced endocannabinoids might also target astrocytic CB_2_R according to the reported expression of this protein in these cells within MS inflammatory lesions [75]. However, these hypotheses remain to be further explored using high-resolution anatomical techniques and cell-type specific endocannabinoid probes [76] applied to the analysis of astrocytes during EAE. Mechanistic considerations notwithstanding, our study adds to the growing body of evidence that supports essential roles for the astrocytic populations of CB_1_R in regulating the astrocyte-endothelial interface in neuroinflammatory disorders.

The exact cascade of events upstream and downstream aCB_1_R signaling that modulate BBB permeability during CNS lesion formation remains a matter of future research in the context of autoimmune inflammation, which should be addressed in clinically relevant in vivo models that enable reliable, high-throughput assessment of astrocytic function at early time points of the inflammatory pathology. According to the existing literature, early-onset activation of aCB_1_R may facilitate BBB dysfunction by attenuating astrocyte reactivity [8, 10]. This possibility is supported by a series of in vivo ablation studies showing that early inhibition of reactive astrogliosis facilitates circulating inflammation to reach the CNS parenchyma and exacerbates clinical disease in the EAE model of MS [77–79]. Some tentative additional support for this hypothesis comes from prior research showing that pharmacological blockade of endocannabinoid hydrolysis inhibits astrocyte reactivity while attenuating disease neuropathology and clinical severity in the EAE and TMEV models of neuroinflammation [80, 81]. However, these in vivo studies did not target CB_1_R as potential underling mechanism and research specifically designed to address the role of CB_1_R in regulating the functions of reactive astrocytes is still scarce. In this context, it is worth mentioning that the protective phenotype of astrocyte-specific CB_1_R null mice in the EAE model that we report here is consistent with recent in vivo observations that aCB_1_-KO mice display significantly less severe neuronal death following induction of global ischemia [82].

To summarize, we propose that early, endogenous activation of CB_1_R in astrocytes drives the pathogenicity of CNS inflammatory lesions by mechanistic interactions that promote BBB permeability and recruitment of immune cells. This study challenges the traditional neuroprotective role of endocannabinoids in MS and adds to the accumulating evidence that points to relevant roles of endocannabinoid signaling via aCB_1_R in the neurovascular adaptations that shape neuroinflammation.

### Limitations of the study

The present study has some limitations to consider. First, like most studies addressing astrocyte-mediated functions of CB_1_R in complex systems, the loss of function approach is based on the expression of Cre recombinase under the control of the human GFAP promoter. Of note, the pattern of recombination efficiency in the tamoxifen GFAP-CreERT2 reporter mouse line, which reflects the transgenic expression level of the human GFAP promoter, varies between brain regions in a similar manner as the intrinsic reporter [37]. Indeed, mouse astrocytes express heterogeneous levels of GFAP, as defined by single-cell transcriptional profiling [83–85]. According to these evidences, astrocyte populations in aCB_1_-KO mice are heterogeneous in terms of Cre-LoxP recombination and deletion of the *Cnr1* gene, which limits the biological interpretation of our results. This technical pitfall, together with the fact that astrocytes express very low levels of CB_1_R as compared to presynaptic elements [31, 86], limits the reliable detection of *Cnr1* gene expression variations between aCB_1_-KO and aCB_1_-WT mice using astrocyte preparations purified based on the expression of cell-surface markers [87]. However, the aCB_1_-KO mouse strain used in this study has been well characterized in terms of aCB_1_R downregulation throughout the CNS using high-resolution electron microscopy and functional approaches [29, 31, 32, 38, 86, 88]. Further, aCB_1_-KO mice present a plethora of behavioral phenotypes that include memory impairments [38], defects in social transmission of stress [89] and protection from stroke [82], as well as resistance to the amnesic [29] and anti-tremor [32] effects of cannabinoids. In the context of these previous evidences, our data state the principle that CB_1_R expressed by astrocytes can exacerbate CNS inflammation.

Second, the low perfusion volume prior to dissection of spinal cord tissue for gene expression analysis may potentially limit the confidence in the results presented. In this study, EAE mice for RT-PCR analysis were perfused with an average of 5 mL of perfusion solution, which makes 3–4 times total blood volume in animals weighing 15–20 g. This approach differs from standardized procedures - typically using 10–20 mL of perfusate - in studies aiming to remove intravascular blood cells prior to CNS analysis. However, to the best of our knowledge there are no direct comparative studies of perfusion volume for capillary cell removal in the EAE model that take into account the remarkable loss of body weight during the acute phase of the disease, and most methodological choices are guided by general CNS perfusion practices. With regard to the gene expression results presented in Figs. 1d and 4a, it should be borne in mind that variability is an intrinsic feature of the EAE model. Both aCB_1_-KO and aCB_1_-WT mice presented marked interindividual differences in terms of disease severity and dispersion in the expression of inflammatory genes within each group is not surprising. Therefore, although we cannot fully exclude the presence of residual blood cells in CNS tissue we are confident that our gene expression results in EAE mice are not limited by technical pitfalls related to inadequate perfusion.

## Supplementary Information

Supplementary Material 1.

Supplementary Material 2.

## Data Availability

The datasets generated during the current study are available from the corresponding authors on reasonable request.

## Abbreviations

aCB_1_R: Astrocyte cannabinoid type-1 receptor
BBB: Blood-brain barrier
CB_1_R: Cannabinoid type-1 receptor
CDH-5: Cadherin 5
cDNA: Complementary DNA
CLN-1: Claudin 1
CLN-4: Claudin 4
CLN-5: Claudin 5
CNS: Central nervous system
FBS: Fetal bovine serum
GFAP: Glial fibrillary acidic protein
ICAM-1: Intercellular adhesion molecule 1
LED: Light-emitting diode
LFB: Luxol fast blue
LPC: Lysophosphatidylcholine
MBP: Myelin basic protein
MOG: Myelin oligodendrocyte glycoprotein
MS: Multiple sclerosis
NDS: Normal donkey serum
NGS: Normal goat serum
OPC: Oligodendrocyte precursor cell
PBS: Phosphate buffered saline
PCR: Polymerase chain reaction
PODXL: Podocalyxin
RIPA: Radioimmunoprecipitation assay
RNA: Ribonucleic acid
RT: Room temperature
TBS: Tris buffer saline
THC: Δ^9^-tetrahydrocannabinol
VCAM-1: Vascular cell adhesion protein 1
VEGF-A: Vascular endothelial growth factor A
ZO-1: Zonula occludens
