# Supplementary material for: Astrocyte CB1 receptors drive blood-brain barrier disruption in central nervous system inflammatory disease
Source: J Neuroinflammation. 2026 Jan 29;23:73. doi: 10.1186/s12974-026-03708-3 (PMC12924220; doi:10.1186/s12974-026-03708-3)
Supplement: Supplementary file 1 — Supplementary Material 1. [file 12974_2026_3708_MOESM1_ESM.pdf]

Western blot Figure 3e: CLN-4

1

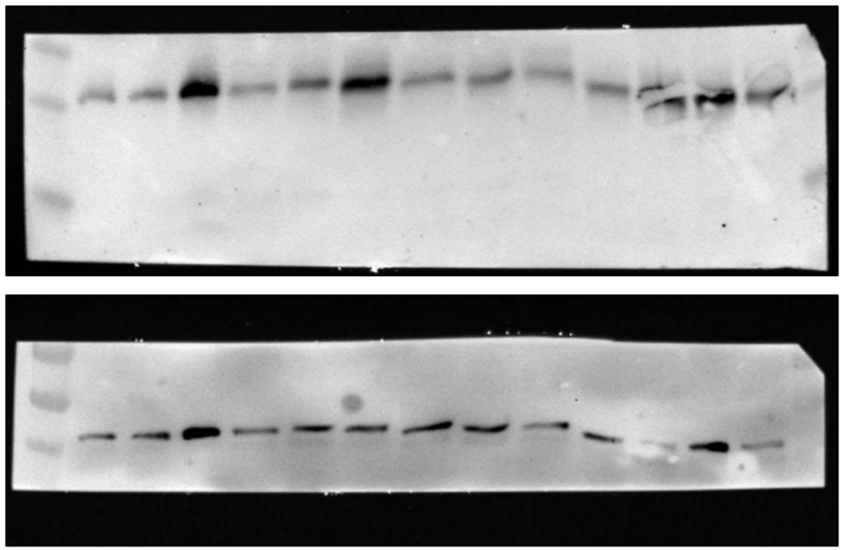

2

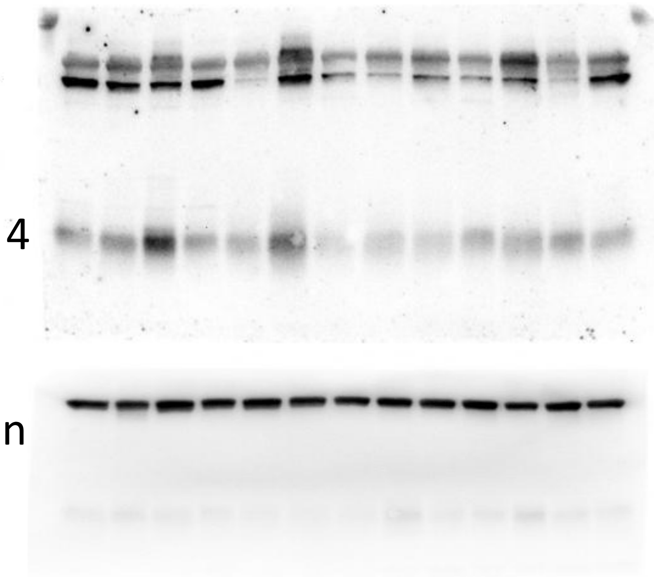

3

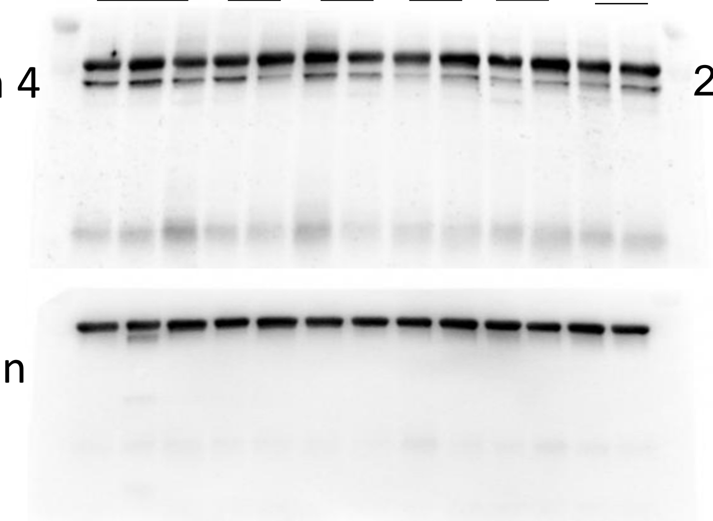

- WT → aCB$_1$-WT
- KO → aCB$_1$-KO

Western blot Figure 4b: ICAM-1

1

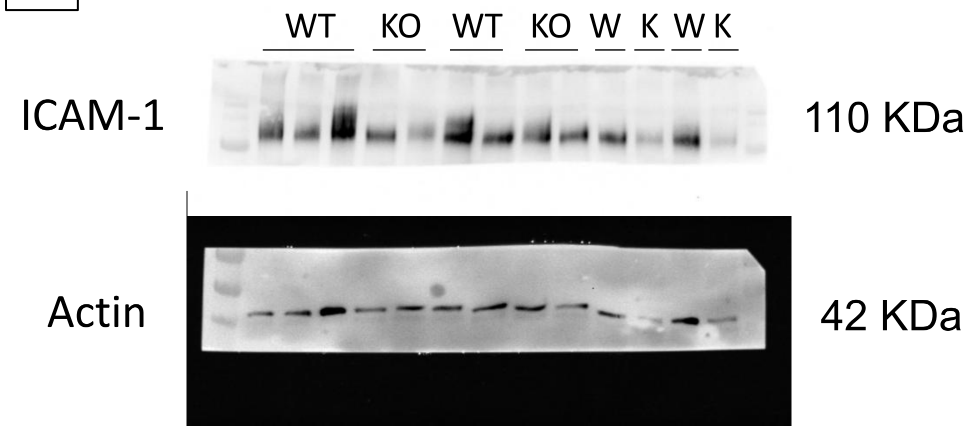

2

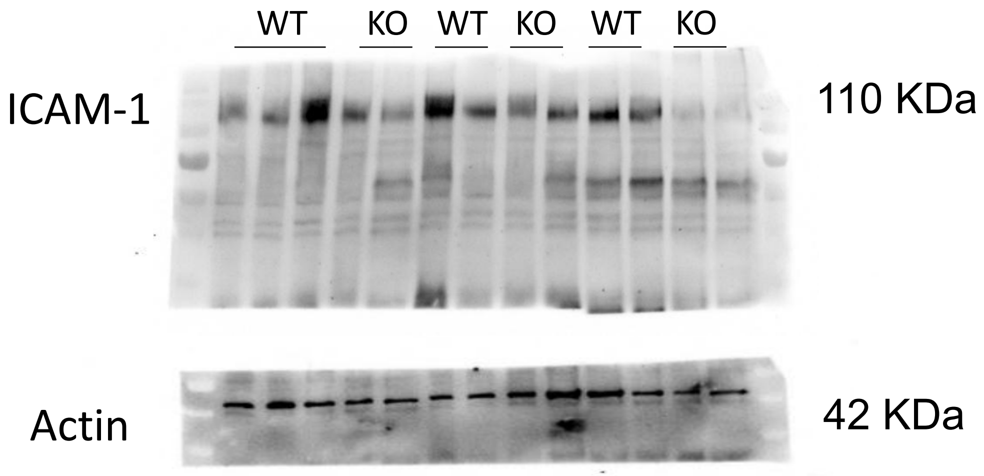

3

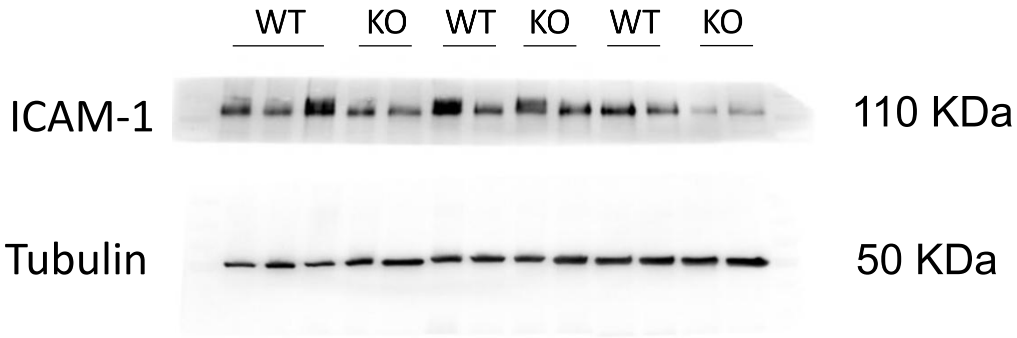

- WT → aCB_1-WT
- KO → aCB_1-KO

Western blot Figure 4b: VCAM-1

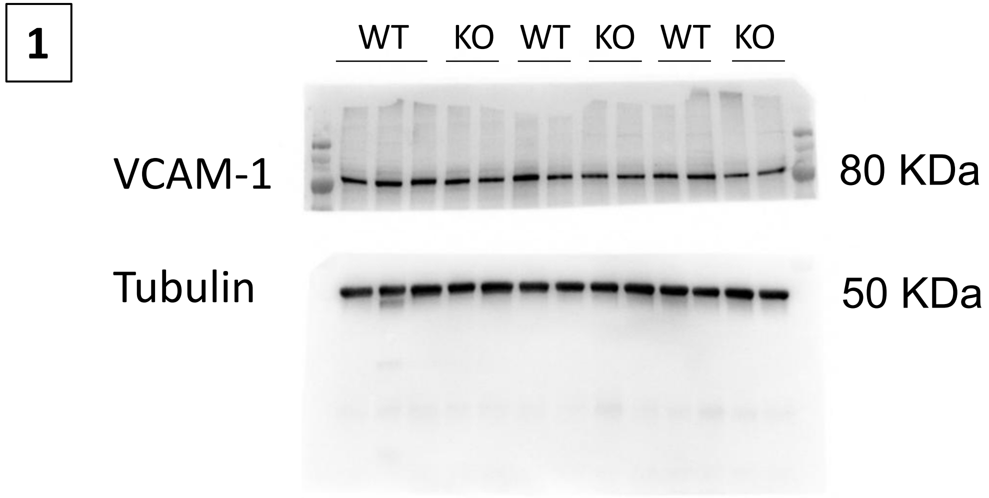

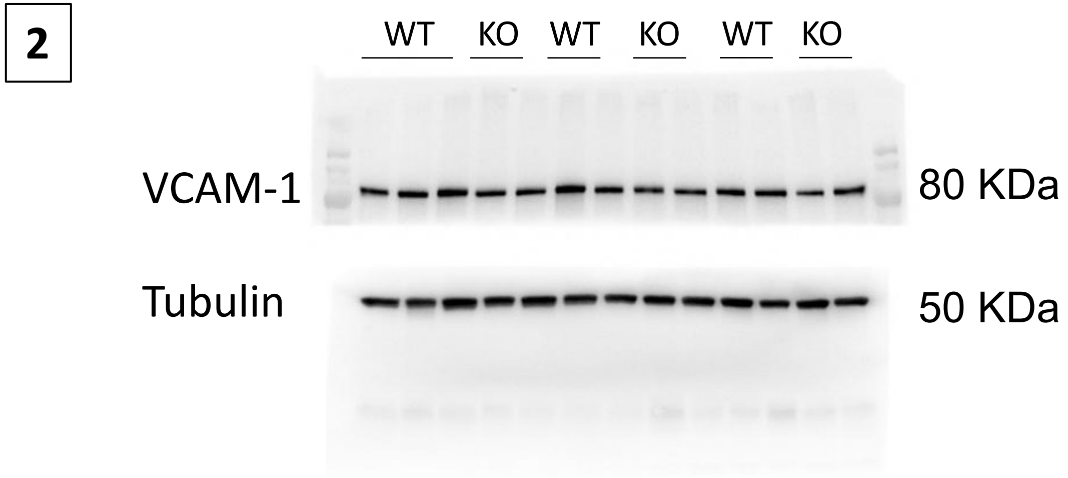

- WT → aCB_1-WT
- KO → aCB_1-KO

Western blot Supplementary Figure 7a: PODXL

1

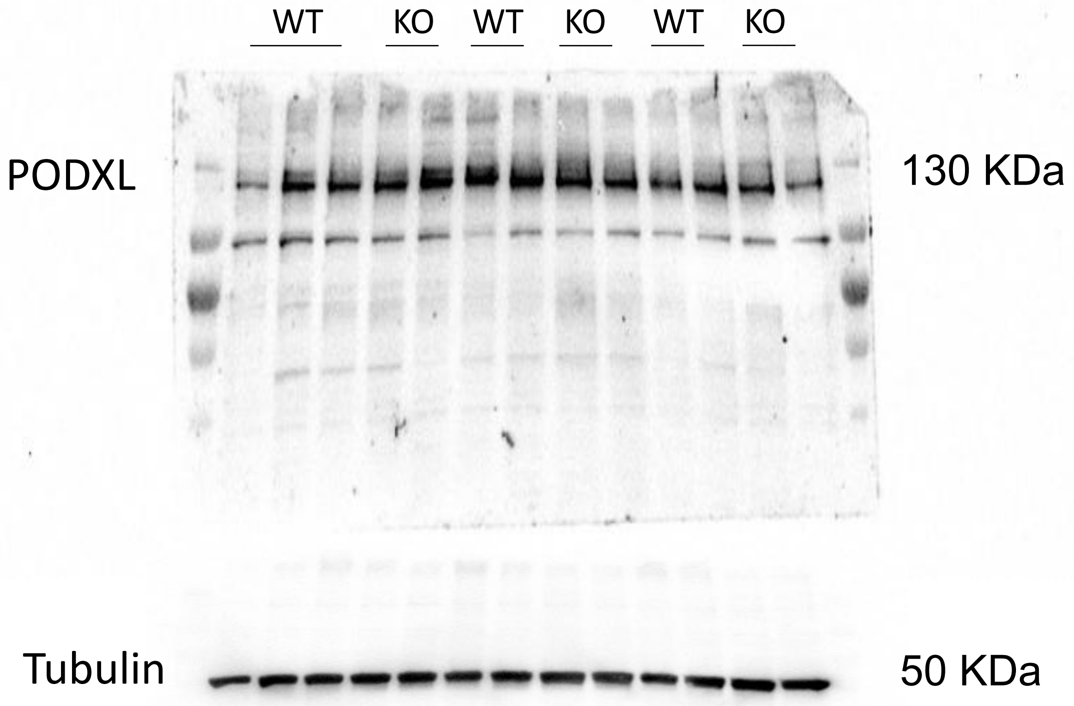

2

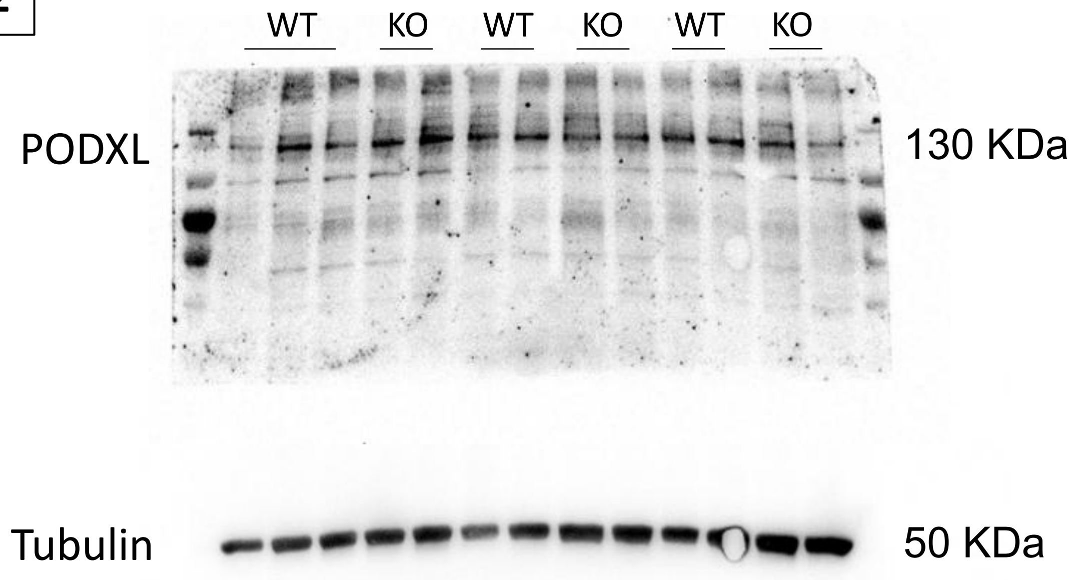

- WT → aCB_1-WT
- KO → aCB_1-KO

Western blot Supplementary Figure 7c: CDH-5

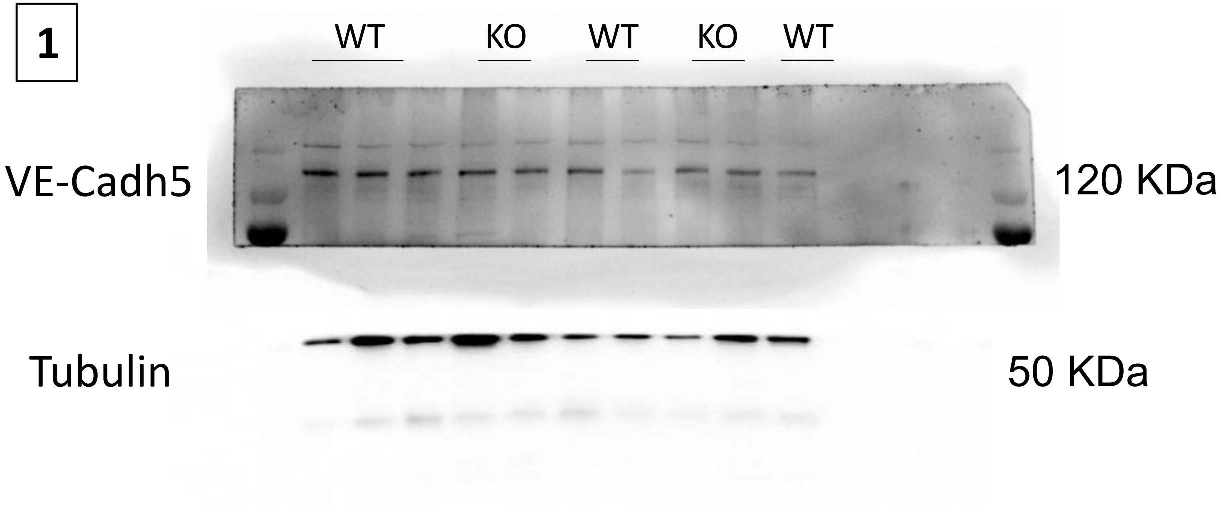

- WT → aCB_1-WT
- KO → aCB_1-KO

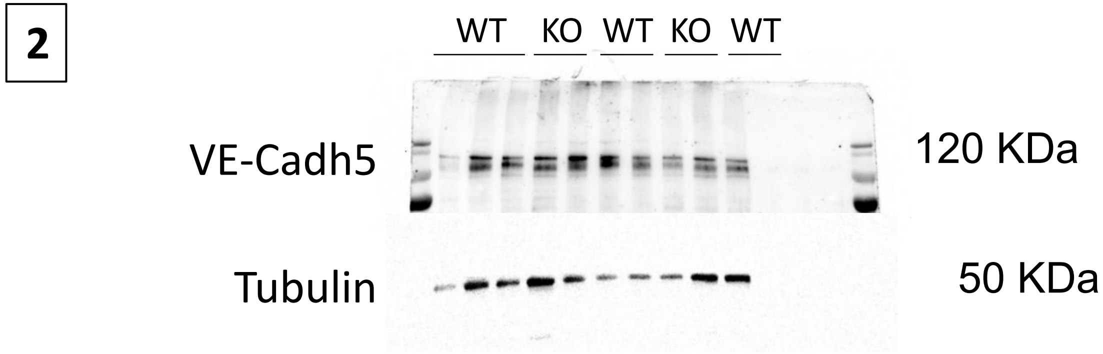

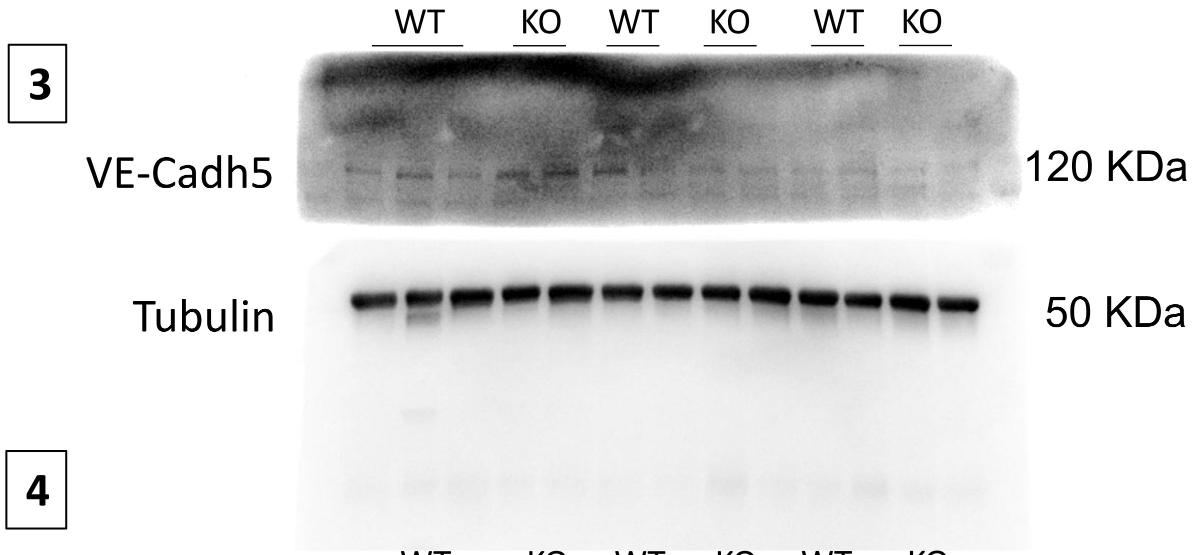

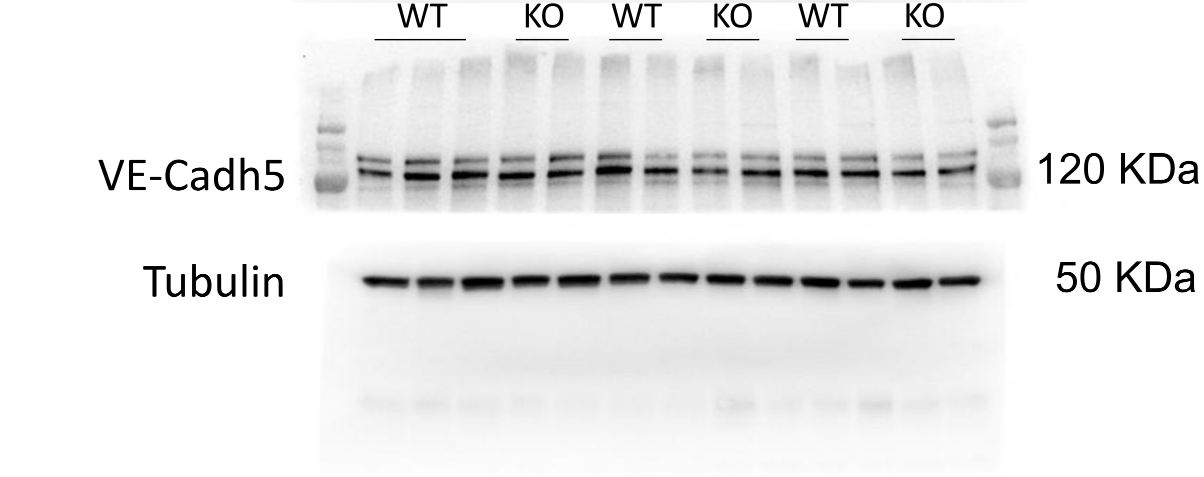

Western blot Supplementary Figure 7c: ZO-1

1

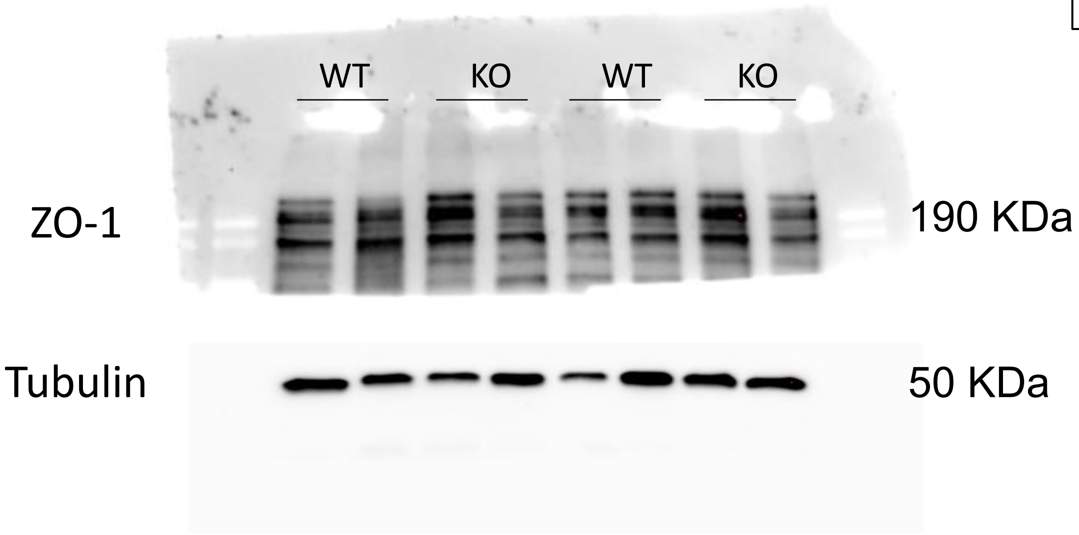

2

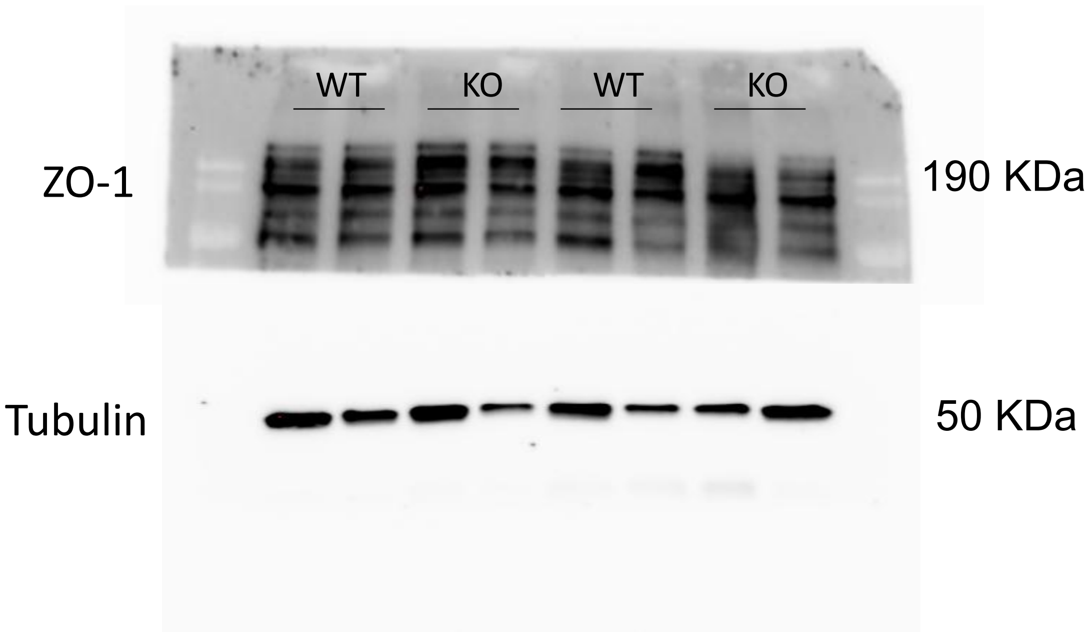

- WT → aCB_1-WT
- KO → aCB_1-KO
