# Supplementary material for: Astrocyte CB1 receptors drive blood-brain barrier disruption in central nervous system inflammatory disease
Source: J Neuroinflammation. 2026 Jan 29;23:73. doi: 10.1186/s12974-026-03708-3 (PMC12924220; doi:10.1186/s12974-026-03708-3)
Supplement: Supplementary file 2 — Supplementary Material 2. [file 12974_2026_3708_MOESM2_ESM.docx]

**Supplementary Figure 1.** (**a**) PCR-based strategy for the analysis of recombination in aCB_1_-WT and aCB_1_-KO mice. The diagram represents the wild-type, floxed, and Cre-recombined *Cnr1* alleles, indicating the position of the primers used for genotyping and PCR amplicon sizes for each reaction. (**b**) Detection of the excised fragment as a result of Cre-mediated recombination in forebrain and spinal cord lysates. Probe hybridization, amplification and electrophoresis resulted in a 689 bp product corresponding to the excision in forebrain and spinal cord tissues from tamoxifen-treated aCB_1_-KO (CB_1_^f/f;GFAP-CreERT2^) mice that was absent in samples from aCB_1_-WT (CB_1_^f/f^) mice. The ~2900 bp amplicon for the unrecombined allele was enriched in lysates from aCB_1_-WT mice. DNA samples with germinal deletion (GD) of the *Cnr1* gene were used as control for recombination. (**c**) Genotyping of the floxed allele rendered a ~595 bp product in aCB_1_-WT and aCB_1_-KO samples and a ~475 bp product in samples from wild-type (WT) mice. The presence of Cre recombinase generated a specific band with 300 bp in aCB_1_-KO samples. DNA samples from Cre^+^ and Cre^-^ mice were run as positive and negative controls.

**Supplementary Figure 2.** Restricted inflammatory neuropathology in aCB_1_-KO mice at acute EAE disease. (**a**) Representative images of spinal cord lesions (*) stained with H&E. Scale bar = 200 µm. The bar graph depicts the quantitative analysis of inflammatory and demyelinating lesions identified in lumbar spinal cord sections from aCB_1_-KO and aCB_1_-WT mice at acute EAE using H&E staining and MBP/Hoechst immunofluorescence, respectively (*n* = 6 mice). (**b**, **c**) Confocal micrographs and quantification of (**b**) GFAP and C3, and (**c**) Iba1 immunostaining in spinal cord demyelinating lesions from aCB_1_-KO and aCB_1_-WT mice normalized to white matter tissue area (*n* = 6 mice). Scale bar = 100 µm. (**d**) Representative images and analysis of GFAP and C3 expression in somatosensory cortex layers V-VI from in aCB_1_-KO and aCB_1_-WT mice (*n* = 8-9 mice). Scale bar = 25 µm. **p* < 0.05 and ***p* < 0.01, unpaired *t*-test or Mann-Whitney test. Error bars express SEM.

**Supplementary Figure 3.** Reduced clinical severity and spinal cord pathology of chronic EAE in astrocyte-specific CB_1_R null mice. (**a**) Clinical scores of aCB_1_-KO and aCB_1_-WT mice during chronic EAE. Comparison of motor scores from symptom onset to 35 dpi revealed significantly attenuated neurological disability in aCB_1_-KO mice. Data are representative of 2 independent EAE experiments pooled together (*n* = 8-12 mice; ****p* < 0.0001; Wilcoxon matched-pairs signed rank test for the comparison of score curves from the onset of EAE symptoms at 6 dpi to 35 dpi). (**b**) Representative images of luxol fast blue (LFB) myelin staining and quantification of demyelinating lesions (*) in spinal cord sections from aCB_1_-KO and aCB_1_-WT mice at 35 dpi (*n* = 8-9 mice). Scale bar = 50 µm. (**c**) Confocal micrographs depict spinal cord sections from aCB_1_-KO and aCB_1_-WT mice double immunostained for MBP and SMI32, GFAP or Iba1. (**d**) Quantification of

MBP immunostaining shows a reduced proportion of demyelinated tissue normalized to white matter area in aCB_1_-KO mice. (**e**) Attenuated neuroaxonal damage (*left panel*) and astrocyte reactivity (*middle panel*), and lower numbers of microglia/macrophages (*right panel*) within inflammatory lesions from aCB_1_-KO mice as quantified by measuring SMI32 or GFAP immunopositive areas and Iba1^+^ cells (*n* = 7-9 mice). Scale bar = 100 µm. **p* < 0.05, ***p* < 0.01 and ****p* < 0.001, unpaired *t*-test or Mann-Whitney test. Error bars express SEM.

**Supplementary figure 4**. Comparative analysis of cortical astrocyte calcium responses in control aCB_1_-WT and aCB_1_-KO mice. The graph depicts the amplitude of sensory-evoked astrocyte calcium signals recorded from non-immunized animals (*n* = 8-12 mice) during 5 non-consecutive days that correspond to 12, 14, 17, 19 and 21 dpi in EAE mice tested in parallel. Analysis using two-way ANOVA followed by Šídák's test for multiple comparisons did not render significant differences between aCB_1_-WT and aCB_1_-KO mice.

**Supplementary figure 5**. Astrocyte-encoded CB_1_R do not modulate oligodendrocyte populations in toxin induced remyelinating lesions. (**a**) Analysis of aCB_1_-KO and aCB_1_-WT mice in the LPC model of toxic demyelination. LPC lesions were analyzed at 14 dpl corresponding to the peak of oligodendrocyte differentiation and the onset of remyelination. Data are representative of 2 independent LPC experiments pooled together. (**b**) Immunohistochemistry for MBP depicts demyelinated lesions (dashed lines) in the *dorsal funiculus* of LPC-injected mice. Scale bar = 50 µm. Quantitative analysis of lesion area shows no differences between genotypes (*n* = 4-7 mice). (**c**, **d**) Quantification of CC1^+^/OLIG2^+^ oligodendrocytes and CC1^-^/OLIG2^+^ immature oligodendroglia in LPC lesions indicates equal cell populations in aCB_1_-KO and aCB_1_-WT animals (*n* = 4-7 mice). Figure **e** shows representative micrographs of LPC lesions triple labelled for MBP, OLIG2 and CC1. Scale bar = 40 µm. (**f**, **g**) Confocal images of LPC lesions double stained for MBP and (**f**) PDGFRα or (**g**) BCAS1. Analysis of PDGFRα^+^ OPCs and BCAS1^+^ myelinating oligodendrocytes in lesions from aCB_1_-KO and aCB_1_-WT mice shows no variations between genotypes (*n* = 3-7 mice). Scale bars = 40 µm. (**h**, **j**) Representative microphotographs and quantitative analysis of (**h**) astrocyte reactivity, (**i**) microglia/macrophage numbers and (**j**) infiltrating inflammatory cells in LPC lesions from aCB_1_-KO and aCB_1_-WT mice double labelled for MBP and GFAP, Iba1 or CD45, respectively. Scale bars = 40 µm. Error bars express SEM.

**Supplementary figure 6**. Comparative analysis of splenic lymphoid and myeloid populations in naive aCB_1_-WT and aCB_1_-KO mice. (**a**) Flow cytometry gating strategy for immune cell analysis presents representative dot-plots from an aCB_1_-WT mouse. (**b**) The total number of splenocytes was similar between genotypes, as were the proportions of (**c**) global T cells (CD3^+^), T helper cells (CD3^+^ CD4^+^), cytotoxic T cells (CD3^+^ CD8^+^), B cells (CD3^-^ CD19^+^) and (**d**) activated T cells (early stage, CD3^+^ CD4^+^/CD8^+^ CD69^+^; late stage CD3^+^ CD4^+^/CD8^+^ CD25^+^). Likewise, the percentages of (**e**) global myeloid cells (CD11b^+^), (**f**) inflammatory monocytes (iMos; CD11b^+^ Ly6C^hi^ Ly6G^-/low^), patrolling monocytes (pMos; CD11b^+^ Ly6C^int^ Ly6G^-^), neutrophils (CD11b^+^ Ly6C^int^ Ly6G^hi^), and classical dendritic cells (cDCs; CD11b^+^ CD11c^+^ Ly6C^-^) showed no differences between aCB_1_-WT and aCB_1_-KO mice (*n* = 4-5 mice). Error bars express SEM.

~~~~

**Supplementary figure 7**. Analysis of the vascular endothelium in EAE lesions from mice lacking astrocyte-encoded CB_1_R. (**a**, **b**) Immunoblotting and/or morphometry analysis of the endothelial cell markers PODXL and laminin in spinal cord tissue from aCB_1_-KO mice and control aCB_1_-WT mice at acute EAE disease (*n* = 6 mice). Scale bars = 50 µm (PODXL) and 100 µm (laminin). (**c**, **d**) Expression of endothelial TJ proteins CDH-5, ZO-1 and CLN-5 in spinal cord tissue from aCB_1_-KO and aCB_1_-WT mice was determined by (**c**) immunoblotting and/or (**d**) immunofluorescence (*n* = 6 mice). Scale bar = 25 µm.

**Supplementary figure 8**. Reduced expression of vascular effector molecules in EAE lesions from astrocyte-specific CB_1_R null mice. (**a**, **b**) Representative confocal micrographs and morphometry show lower expression levels of the adhesion molecules ICAM-1, VCAM-1 and VEGF-A within lesion (L) and perilesion (PL) areas from aCB_1_-KO mice (*n* = 6 mice). Scale bar = 100 µm. **p* < 0.05 and ***p* < 0.01, unpaired *t*-test or Mann-Whitney test. Error bars express SEM.

**Supplementary Table 1. Antibodies for flow cytometry**

| Antibody | Clone | Concentration | Reference |
| --- | --- | --- | --- |
|  |  |  |  |
| CD3e-PB | 500A2 | 0.2 µg/10^6^ cells | BD Biosciences (#558214) |
| CD4-PE | RM4-5 | 0.1 µg/10^6^ cells | BD Biosciences (#553049) |
| CD8a-FITC | 53-6.7 | 0.25 µg/10^6^ cells | BD Biosciences (#553031) |
| CD11b-PerCP-Cy5.5 | M1/70 | 0.2 µg/10^6^ cells | BD Biosciences (#550993) |
| CD11c-APC | N418 | 0.2 µg/10^6^ cells | Thermo Fisher (#17-0114-82) |
| CD19-PE | 6D5 | 0.025 µg/10^6^ cells | BioLegend (#115508) |
| CD25-Pe-Cy5.5 | PC61.5 | 0.2 µg/10^6^ cells | Thermo Fisher (#35-0251-82) |
| CD69-APC | H1.2F3 | 0.2 µg/10^6^ cells | Thermo Fisher (#17-0691-82) |
| Ly6C-FITC | AL-21 | 0.2 µg/10^6^ cells | BD Biosciences (#553104) |
| Ly-6G-PE | 1A8  A81 | 0.2 µg/10^6^ cells | BD Biosciences (#551461) |
|  |  |  |  |
|  |  |  |  |

**Supplementary Table 2. Primers for RT-qPCR analysis**

| **Gene** | **Forward primer** | **Reverse primer** |
| --- | --- | --- |
|  |  |  |
| *B2m* | ACTGACCGGCCTGTATGCTA | ATGTTCGGCTTCCCATTCTCC |
| *Bdnf* | TCCAAAGGCCAACTGAAGCA | CTGCAGCCTTCCTTGGTGTA |
| *C1ra* | CCTTCCACCCCATAGAACAG | CCTTCAACTTACAGTGCCTGA |
| *C3* | AGGGAGTGTTTGTGCTGAAC | GCCAATGTCTGCCTTCTCTAC |
| *Ccl2* | GTAGTTTTTGTCACCAAGCTC | CAGAAGTGCTTGAGGTGGTT |
| *Ccl5* | GCTCCAATCTTGCAGTCGT | CCTCTATCCTAGCTCATCTCCA |
| *Cldn1* | ACAGCATGGTATGGAAACAGA | AGGAGCAGGAAAGTAGGACA |
| *Cldn4* | CACTCAGCCTACACGTTACTC | CACTCAGCACACCATGACTT |
| *Cntf* | GTGAAGACAGAAGCAAACCAG | AGATAGAGCGGCTACAGA GG |
| *Cxcl10* | ATTTTCTGCCTCATCCTGCT | TGATTTCAAGCTTCCCTATGGC |
| *Fkbp5* | GACACCAAAGAAAAGCTGACG | ACTATCTTCCTGTACTGA ATC ACG |
| *Gapdh* | AGACGGCCGCATCTTCTT | TTCACACCGACCTTCACCAT |
| *Gbp2* | GCAGAATTCACCTCATACATCTTG | GATGGCACCAACATAGGTCTG |
| *Ggta1* | TCCTGTTGATGCTGATTGTCTC | GTCCTTCTGCCATCTGTT CTC |
| *H2-T23* | ACAGTCCCGACCCAGAGTAG | CCACGTAGCCGACAATGATGA |
| *Hprt* | CAGTACAGCCCCAAAATGGTTA | AGTCTGGCCTGTATCCAACA |
| *Icam1* | CTGTGCTTTGAGAACTGTGG | GGTCCTTGCCTACTTGCTG |
| *Igf1* | ATGCTCTTCAGTTCGTGTGT | AGTACATCTCCAGTCTCC TCA G |
| *Iigp1* | CTCATGTGAAGAGCCTGTAGC | CTGACCCATGACTTCAAGCA |
| *Mbp* | CCCTCACAGCGATCCAAGTA | CTCTGTGCCTTGGGAGGAA |
| *Mog* | CACTTGTGCCTACGATCCTC | AGTCCGATGGAGATTCTCTACT |
| *Ntf3* | AGTCCACCTTTCTCTTCATGTC | ACATCACCTTGTTCACCTGTA |
| *Olig2* | AGGGATGATCTAAGCTCTCGAA | ATTACAGACCGAGCCAAC AC |
| *Pdgfa* | ATTAACCATGTGCCCGAGA | GTATCTCGTAAATGACCGTCC TG |
| *Pdgfra* | TCACAGCCACCTTCATTACAG | GTTGCCTTACGACTCCAGATG |
| *Ppia* | AGGGTTCCTCCTTTCACAGAA | TGCCGCCAGTGCCATTA |
| *Psmb8* | GCTGCTTTCCAACATGATGC | CCGAGTCCCATTGTCATC TAC |
| *Serping1* | CTACCAAGATGGCTAAGACCAA | GATGCTCTCCAAGTTGCT CT |
| *Tgfb1* | GCTGCGCTTGCAGAGATTAA | GTAACGCCAGGAATTGTTGCTA |
| *Vcam1* | GCAAAGGACACTGGAAAAGAG | TGTGCAGTTGACAGTGACA |
| *Vegfa* | AGAAAGACAGAACAAAGCCAGA | TCGTTTAACTCAAGCTGCCT |
|  |  |  |

**Supplementary Table 3. Primary antibodies for immunohistochemistry**

| Antibody | Host | Dilution | Reference |
| --- | --- | --- | --- |
|  |  |  |  |
| Aquaporin-4 | Guinea pig | 1:1000 | Synaptic Systems (#429004) |
| APP | Mouse | 1:200 | Merck (#MAB348) |
| B220 | Rat | 1:200 | BD Biosciences (#557390) |
| BCAS1 | Rabbit | 1:500 | Synaptic Systems (#445003) |
| C3 | Rabbit | 1:1000 | Dako (#A0063) |
| CDH-5 | Goat | 1:100 | R&D Systems (#AF1002) |
| CC1 | Mouse | 1:200 | Calbiochem (#OP80) |
| CCL2 | Mouse | 1:300 | Santa Cruz (#sc-32771) |
| CD3 | Rat | 1:50 | Bio-Rad (#MCA1477) |
| CD45 | Rat | 1:100 | BD Pharmigen, (#550539) |
| CD68 | Rat | 1:100 | Bio-Rad (#MCA1957GA) |
| CLN-1 | Mouse | 1:100 | ThermoFisher (#37-4900) |
| CLN-4 | Mouse | 1:100 | ThermoFisher (#32-9400) |
| CLN-5 | Mouse | 1:100 | Invitrogen (#35-2500) |
| Fibrinogen | Sheep | 1:200 | US Biological (#F4199) |
| GFAP | Rabbit | 1:2000 | Dako (#(#Z0334) |
| GFAP | Chicken | 1:1000 | Abcam Ab4674) |
| Iba1 | Rabbit | 1:500 | Wako (#019-19741) |
| ICAM-1 | Goat | 1:500 | R&D Systems (#AF796) |
| Laminin | Rabbit | 1:500 | Merck (#L9393) |
| Ly6G | Rat | 1:100 | BioLegend (#127601) |
| MBP | Chicken | 1:200 | Merck (#ab9348) |
| MBP | Mouse | 1:1000 | Biolegend (#808402) |
| NG2 | Rabbit | 1:200 | Merck (#AB5320) |
| Olig2 | Mouse | 1:500 | Merck (#MABN50) |
| PDGFRα | Goat | 1:250 | R&D Systems (#AF1062) |
| PODXL | Goat | 1:500 | R&D Systems (#AF1556) |
| SMI32 | Mouse | 1:200 | BioLegend (#801702) |
| VCAM-1 | Rabbit | 1:500 | Abcam (#ab134047) |
| VEGF-A | Rabbit | 1:500 | Santa Cruz (#sc-507) |
|  | Mouse | 1:500 | Santa Cruz (#sc-7269) |
| ZO-1 | Rabbit | 1:100 | Invitrogen (#402200) |
|  |  |  |  |
